# Autophagy in Multiple Sclerosis: Two Sides of the Same Coin

**DOI:** 10.3389/fncel.2020.603710

**Published:** 2020-11-20

**Authors:** Chairi Misrielal, Mario Mauthe, Fulvio Reggiori, Bart J. L. Eggen

**Affiliations:** ^1^Molecular Neurobiology, Department of Biomedical Sciences of Cells and Systems, University Medical Center Groningen, University of Groningen, Groningen, Netherlands; ^2^Molecular Cell Biology, Department of Biomedical Sciences of Cells and Systems, University Medical Center Groningen, University of Groningen, Groningen, Netherlands

**Keywords:** autophagy, multiple sclerosis, neurodegeneration, inflammation, resolution

## Abstract

Multiple sclerosis (MS) is a complex auto-immune disorder of the central nervous system (CNS) that involves a range of CNS and immune cells. MS is characterized by chronic neuroinflammation, demyelination, and neuronal loss, but the molecular causes of this disease remain poorly understood. One cellular process that could provide insight into MS pathophysiology and also be a possible therapeutic avenue, is autophagy. Autophagy is an intracellular degradative pathway essential to maintain cellular homeostasis, particularly in neurons as defects in autophagy lead to neurodegeneration. One of the functions of autophagy is to maintain cellular homeostasis by eliminating defective or superfluous proteins, complexes, and organelles, preventing the accumulation of potentially cytotoxic damage. Importantly, there is also an intimate and intricate interplay between autophagy and multiple aspects of both innate and adaptive immunity. Thus, autophagy is implicated in two of the main hallmarks of MS, neurodegeneration, and inflammation, making it especially important to understand how this pathway contributes to MS manifestation and progression. This review summarizes the current knowledge about autophagy in MS, in particular how it contributes to our understanding of MS pathology and its potential as a novel therapeutic target.

## Introduction

Autophagy is a lysosomal degradation system for damaged or unwanted organelles, aggregates, and long-lived proteins, which is important for cellular homeostasis (Choi et al., 2013). This process is responsible for nutrient supply under starved conditions by recycling the metabolites composing cellular components (Lahiri et al., 2019). Autophagy is also involved in a multitude of other physiological functions, including the regulation of innate and adaptive immune responses (Beau et al., 2011; Levine et al., 2011). In recent years, the involvement of autophagy in several pathological conditions, such as neurodegenerative disorders and autoimmune diseases, has become evident as well (Mizushima et al., 2008; Law et al., 2010; Ravikumar et al., 2010; van Beek et al., 2018; Yin et al., 2018; Levine and Kroemer, 2019).

Multiple sclerosis (MS) is a demyelinating auto-immune disorder of the central nervous system (CNS), which is driven by a complex interaction between environmental, genetic, and immunological factors. MS is characterized by the interplay of neuroinflammatory and neurodegenerative processes, resulting in progressive disability of patients (Dyment et al., 2006; Sawcer et al., 2011; Dobson and Giovannoni, 2019). Although this disease has been viewed for a long time as a T-cell-mediated autoimmune disease, recent investigations have uncovered that MS is a complex disorder that involves many cell types, including both other immune cells, such as dendritic and B-cells, and CNS cells, including neurons and glial cells. Most patients suffer from a relapsing-remitting disease course that is characterized by bouts of inflammation and neurodegeneration, which eventually transitions into progressive MS (Dobson and Giovannoni, 2019). Yet, the precise molecular causes underlying MS as well as the mechanisms driving either relapsing-remitting or progressive disease progression, remain largely unknown. There is no cure for MS and current treatments are mainly focused on the relapsing-remitting phase of the disease and they primarily target the immune system.

In this review, the function of autophagy in regulating neuroinflammation and neurodegeneration in MS is discussed, with a particular focus on how autophagy interferes with the regulation and functioning of different cell types that contribute to the pathophysiology of this devastating disease.

## The Regulation and Mechanism of Autophagy

Different types of autophagy have been described based on their differences in regulation, type of cargo, and the lysosomal delivery mechanism: chaperone-mediated autophagy, microautophagy, and macroautophagy (Feng et al., 2018). These processes are described in detail elsewhere (Martinez-Vicente and Cuervo, 2007; Cuervo, 2010; Li et al., 2012; Feng et al., 2014) and here we focus on the regulation of macroautophagy since this process is best described in brain disorders (Nixon, 2013; Liang and Le, 2015; Menzies et al., 2017; van Beek et al., 2018; Yin et al., 2018; Levine and Kroemer, 2019; Stamatakou et al., 2020).

Macroautophagy, hereafter referred to as autophagy, is characterized by the sequestration of cytoplasmic substrates by double-membrane vesicles called autophagosomes, which originates from membranous cisterna, the phagophores, generated *de novo* upon autophagy induction. Completed autophagosomes then fuse with lysosomes to deliver their cargo in the interior of this hydrolytic organelle. The metabolites resulting from the degradation of the autophagosomal cargoes are recycled back to the cytosol for the synthesis of new proteins or are used for the generation of energy (Lahiri et al., 2019).

**FIGURE 1:**
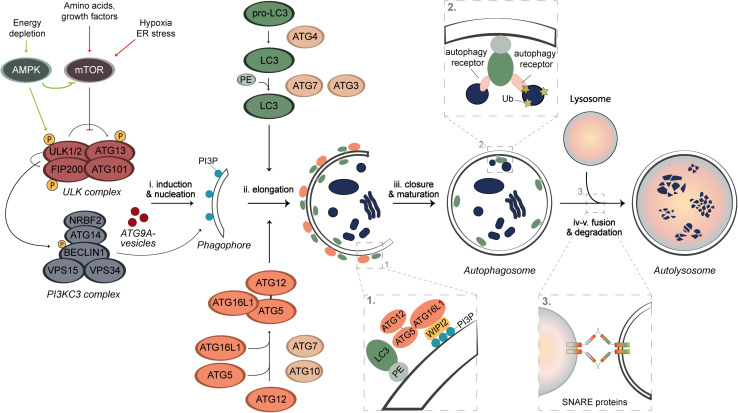
Schematic overview of autophagy. Under nutrient rich conditions, the autophagy process is negatively regulated by mTORC1, whose activity can be inhibited through AMPK activation, starvation, hypoxia, or stress. The latter lead to a de-repression of the ULK kinase complex, which self-activates through autophosphorylation and stimulates the recruitment and activation of the PI3KC3 complex. PI3KC3 produces PI3P on the phagophore membrane, which is needed for the recruitment of the ubiquitin complexes to the membrane of the autophagosome. The nucleation is mediated by ATG9A vesicles, ULK, and PI3KC3 complexes. The autophagosome formation requires two Ub-like conjugation systems. LC3 proteins are post-translationally processed by ATG4 proteases and upon induction of autophagy, they are activated by ATG7 and ATG3 enzymes and conjugated with PE. This event is guided by a complex formed by the second Ub-like conjugation system. ATG7 activates ATG12, which is then covalently linked to ATG5 by ATG10 and subsequently associates with ATG16L1 to form a multimeric complex. This complex is anchored onto the phagophore by interacting with the PI3P effector protein WIPI2. The proteins on the external surface dissociate after completion, whereas LC3-PE permanently integrates on the internal surface of the membrane. The complete autophagosome ultimately fuses with lysosomes in a SNARE-mediated manner to form an autolysosome, in which the autophagosomal cargo is degraded by lysosomal enzymes.

Autophagy is a highly conserved and dynamic process that can be subdivided into five sequential steps; (i) induction and nucleation of the phagophore, (ii) phagophore elongation, (iii) phagophore closure and autophagosome maturation, (iv) autophagosome fusion, and (v) cargo degradation (Figure 1). These steps involve a cascade of events that are mediated by proteins, most of which have been named as autophagy-related (ATG) proteins (Figure 1; Nakatogawa, 2020). Upon autophagy induction, the ULK kinase complex, which consists of the serine/threonine kinases ULK1 or ULK2, FIP200, ATG13, and ATG101, gets activated through self-phosphorylation and stimulates the formation of the class III phosphatidylinositol 3-kinase (PI3KC3) complex (Nakatogawa, 2020). The PI3KC3 complex consists of the BECLIN1, VPS34, VPS15, ATG14, and NRBF2 subunits, and generates phosphatidylinositol 3-phosphate (PI3P) on the phagophore membrane (Nakatogawa, 2020). PI3P is key for the recruitment of several downstream ATG proteins that bind to this lipid, such as WIPI2 (Qian et al., 2017). Together with the ULK complex and ATG9A-positive vesicles, PI3KC3 catalyzes the nucleation of the phagophore (Figure 1; Nakatogawa, 2020). The elongation process involves two ubiquitin (Ub)-like conjugation systems that is composed by several ATG proteins. The first system involves the activation of ATG12 by ATG7 which is then transferred via ATG10 to ATG5 to generate the ATG12-ATG5 conjugate, which associates to ATG16L1. This is then recruited to the phagophore membrane by WIPI2, forming a multimeric complex (Figure 1; Nakatogawa, 2020). In parallel, ATG7, ATG4, and ATG3 are involved in another system that is responsible for the conjugation of LC3 proteins to phosphatidylethanolamine (PE). This conjugation occurs on the phagophore membrane and is guided by the ATG12-ATG5-ATG16L1 complex (Figure 1; Nakatogawa, 2020). Conjugated LC3 proteins are present on the internal and external surface of the expanding phagophore to mediate the expansion and closure of the autophagosome (Nakatogawa, 2020). Once autophagosomes are completed, they traffic toward lysosomes and fuse with these organelles through an event mediated by SNARE proteins and other fusion co-factors, to form the so-called autolysosomes (Figure 1). After fusion, the content of the autophagosome is exposed to lysosomal enzymes and the metabolites generated by degradation are recycled to the cytosol via permeases on the limiting membrane of lysosomes (Lahiri et al., 2019).

Autophagy can either be non-selective, referred to as bulk autophagy and it is activated, e.g., under starved conditions to recycle cellular components in an apparent random manner, or selective. During selective types of autophagy, damaged or superfluous organelles but also other structures, including mitochondria (mitophagy), lipid droplets (lipophagy), ribosomes (ribophagy), and invading pathogens (xenophagy), are specifically and exclusively sequestered by autophagosomes (Kirkin and Rogov, 2019). The pool of PE-conjugated LC3 proteins in the inner surface of phagophores promote the cargo engulfment via LC3-interacting regions (LIR) that are present on the so-called autophagy receptors, some of which are soluble (e.g., p62/SQSTM1, NDP52, or OPTN) and bind to ubiquitinated cargo, while other are present on organelles (e.g., NIX on mitochondria or FAM134B on the endoplasmic reticulum) (Figure 1; Kirkin and Rogov, 2019). This recognition system allows selective degradation of specific cargo.

Under nutrient-rich conditions, autophagy is negatively regulated by the mammalian target of rapamycin complex 1 (mTORC1) that phosphorylates and inactivates the ULK kinase complex (Figure 1; He and Klionsky, 2009; Zachari and Ganley, 2017). Upon removal of nutrients or energy, autophagy is induced via inhibition of mTORC1 and/or through direct phosphorylation and activation of the ULK kinase complex by adenosine monophosphate-activated protein kinase (AMPK), resulting in the activation of the downstream machinery of autophagy and consequently initiating this process (Figure 1; He and Klionsky, 2009; Puente et al., 2016; Keller and Lünemann, 2017; Zachari and Ganley, 2017). The modulation of these kinases is currently the major strategy to induce autophagy *in vivo* and in patients (Menzies et al., 2017; Djajadikerta et al., 2020).

## Autophagy and Neurodegeneration

A hallmark shared by many neurodegenerative diseases of the CNS is neuronal loss, which can have a range of causes, from the formation of cytotoxic aggregates to mitochondrial dysfunction and/or iron accumulation (Wong and Holzbaur, 2014).

Neurons heavily depend on autophagy for their survival and maintenance of homeostasis (Hara et al., 2006; Komatsu et al., 2006), and therefore it is not surprising that dysfunction of this process causes neurodegenerative diseases (Fujikake et al., 2018). Defects in different steps of the autophagy process, such as impaired autophagosome formation, inhibited autolysosome formation, or disrupted lysosomal function have been observed, e.g., Alzheimer’s disease (AD), Huntington’s disease (HD), and Parkinson’s disease (PD) (Nixon, 2013; Menzies et al., 2017).

Consistently, loss of *Atg7* or *Atg5* in the CNS of mice or neurons causes neurological defects and severe damage to neurons (Komatsu et al., 2006; Cuervo, 2010; Nixon, 2013; Stavoe and Holzbaur, 2019). Autophagy is important to degrade physiological and potentially cytotoxic protein aggregates and has a protective effect against the disease-associated aggregates characterizing AD, HD, and PD (Ravikumar et al., 2004; Nixon and Yang, 2011; Nixon, 2013; Menzies et al., 2017; Fujikake et al., 2018). The conditional deletion of *ATG* genes in mice leads to the accumulation of aggregates (Hara et al., 2006; Komatsu et al., 2006) and progressive neuronal death in different areas of the brain (Menzies et al., 2017). In MS lesions, extracellular aggregates of fibronectin are observed (Stoffels et al., 2013), however, it remains to be determined whether their appearance is connected to a deficient ATG machinery. In addition to aggregate removal, autophagy can degrade damaged mitochondria, which when impaired, can also contribute to neuronal damage and death (Wong and Holzbaur, 2014). Consequently, pharmacological induction of autophagy showed beneficial effects in a wide range of neurodegenerative diseases, such as HD and AD (Menzies et al., 2017).

Besides the intracellular defects in neurons that lead to neuronal damage, external stimuli can also cause neuronal loss. For example, neuroinflammation is often observed in neurodegenerative diseases, where it contributes to neuronal damage (Rubinsztein et al., 2015), and autophagy is emerging as an important modulator of inflammation (discussed below). Moreover, autophagy is critical for debris clearance, and its impairment delays myelin debris clearance after nerve injury (Jang et al., 2016), which prevents efficient remyelination and further leads to neuronal damage and neurodegeneration, which are typical in MS.

## Autophagy and Inflammation

The immune system is essential to maintain systemic health by eliminating pathogens and preventing infections, and damaged cells. The inflammatory response of immune cells plays an essential role in this process and involves many cell types. Autophagy has been implicated in both the innate and adaptive immune response, playing a role in pathogen removal, antigen presentation, cytokine production, lymphocyte survival, and development of specific cell types (Miller et al., 2008; Levine et al., 2011; Shi et al., 2012; Deretic et al., 2013; Qian et al., 2017; Yin et al., 2018). The link between autophagy and inflammation is complex and reciprocal since they can either induce or suppress each other through different mechanisms (Levine et al., 2011; Deretic et al., 2013; Liang and Le, 2015). Therefore, it is not surprising that autophagy has been functionally and/or pathologically connected to several neuroinflammatory diseases, including AD, HD, amyotrophic lateral sclerosis (ALS), and MS (Levine et al., 2011; Muller et al., 2017; Yin et al., 2018).

Autophagy can be induced by different pro-inflammatory stimuli, such as toll-like receptor (TLR) activation, damage-associated molecular patterns (DAMPs), and pathogen-associated molecular patterns (PAMPs) (Harris and Keane, 2010; Levine et al., 2011; François et al., 2014; Liang and Le, 2015; Yin et al., 2018). On the other hand, it can be inhibited by Th2-associated pro-inflammatory cytokines, such as IL-4 and IL-13 (Harris et al., 2007; Harris and Keane, 2010; Park et al., 2011; Deretic et al., 2013). In its turn, autophagy inhibits, for example, the inflammatory IL-1β and IL-18 responses (Shi et al., 2012; Liang and Le, 2015; Zhang H. et al., 2016) by degrading inflammasomes (Shi et al., 2012; Deretic et al., 2013). Further, it also prevents the production of reactive oxygen species (ROS) that activate inflammasomes by eliminating damaged mitochondria (Qian et al., 2017). Overall, autophagy is a negative feedback regulator of the immune system, participating in the resolution of inflammation and returning it to homeostasis (Levine et al., 2011). However, autophagy is also implicated in T-cell survival and polarization, the differentiation and survival of antibody-secreting plasma cells, and the enhancement of antigen presentation in dendritic cells (DCs) (Pengo et al., 2013; Conway et al., 2013; Deretic et al., 2013; Qian et al., 2017), which are all processes that form the core of immune responses. Thus, dysregulation of autophagy can prolong and make persisting inflammatory responses after an insult, possibly leading to autoimmune and inflammatory diseases.

Genome-wide association studies have revealed the connection of several *ATG* genes with inflammatory and autoimmune disorders (Muller et al., 2017). It is important to note that the regulation of autophagy varies in different inflammatory diseases. Pharmacological inducers of autophagy appear to be protective against psoriasis (Varshney and Saini, 2018) and inflammatory bowel disease (Saitoh et al., 2008), whereas inhibition of this process ameliorates illnesses such as systemic lupus erythematosus (Clarke et al., 2015), rheumatoid arthritis (Lin et al., 2013), and MS (Kovacs et al., 2012).

The crosstalk between autophagy and the immune system emphasizes the importance of this process in the pathogenesis of autoimmune disorders, including MS.

## Autophagy and MS

Multiple sclerosis is characterized by inflammation, demyelination, and neurodegeneration, all processes that have been connected to autophagy, and therefore, investigating autophagy in the context of MS is relevant. In blood samples from MS patients, several *ATG* genes involved in multiple steps of the autophagy process were differently expressed; *ATG9A* and *BECN1* were downregulated, while *ULK1, ULK2*, and *ATG5* were upregulated (Igci et al., 2016). In addition, in experimental autoimmune encephalomyelitis (EAE), an MS mouse model, LC3 and BECLIN1 protein levels were reduced while those of p62/SQSTM1 were increased in the spinal cords of these animals. Moreover, inhibition of mTORC1 ameliorated disease severity (Boyao et al., 2019), suggesting that autophagy is negatively affected in EAE mice. Inhibition of autophagy can also result in the accumulation of damaged mitochondria and the production of ROS (Chen et al., 2008; Hassanpour et al., 2020), which both contribute to the demyelination process in MS. Another approach to enhance autophagy is through caloric restriction, where cycles of a fasting-mimicking diet are applied, and this regime has been shown to ameliorate disease severity and stimulates remyelination in both EAE mice and relapsing-remitting MS patients (Choi et al., 2016).

Importantly, a few studies have indicated that autophagy is differently involved in both relapsing and progressive forms of MS. In a cohort study, autophagic activity was increased in relapsing-remitting MS patients (Hassanpour et al., 2020), and ultrastructural analyses revealed the presence of synaptic vesicle-containing autophagosomes in the dentate nucleus from a chronic MS patient (Albert et al., 2017), suggesting a pathological role of autophagy in MS. Treatment with an mTORC1 inhibitor, however, resulted in beneficial effects in both relapsing-remitting EAE mice (Esposito et al., 2010) and patients with MS (Hassanpour et al., 2020). This emphasizes the importance to further elucidate how autophagy is involved in different forms of MS.

Although autophagy is important to maintain homeostasis in all cell types, its requirement for other functions and consequently its regulation varies in the different cell types and consequently its regulation differs as well (Liang and Le, 2015). This aspect also emerges in the context of MS, in which autophagy appears to contribute to the pathology in DCs, T-cells and B-cells, while it has a protective role in neurons and glial cells.

### Dendritic Cells

DCs are the main peripheral antigen-presenting cells (APCs) that can trigger a T-cell response (Nuyts et al., 2013). Antigen presentation is required for both T-cell development and their activation, through the expression of surface molecules and cytokine secretion from DCs (Yogev et al., 2012). DCs are the most efficient APCs for reactivating myelin-specific CD4^+^ T-cells in the CNS (Yogev et al., 2012; Mohammad et al., 2013), and they are present in cerebrospinal fluid (CSF) and CNS lesions of MS patients (Nuyts et al., 2013).

It was hypothesized that removing DCs could inhibit EAE development, however, depletion of DCs in mice showed a stronger inflammatory response and enhanced EAE severity (Yogev et al., 2012). The levels of regulatory T-cells (Treg) were also lower (Yogev et al., 2012; Mohammad et al., 2013), confirming the important role of DCs in regulating T-cell homeostasis. In addition, a study where major histocompatibility complex (MHC) class II expression was only restricted to DCs, revealed that DCs are sufficient to present antigens to T-cells in order to mediate CNS inflammation in EAE mice (Greter et al., 2005). Altogether, these data show that the status of DCs is crucial for MS development, i.e., steady-state DCs play a protective role by inducing self-tolerance and by differentiating Treg cells, whereas activated DCs are responsible for the stronger immunogenic response by activating CD4^+^ T-cells (Greter et al., 2005; Yogev et al., 2012; Mohammad et al., 2013). These observations have raised the question whether the molecular pathway of antigen presentation to CD4^+^ T-cells could be modulated to prevent immune activation.

**FIGURE 2:**
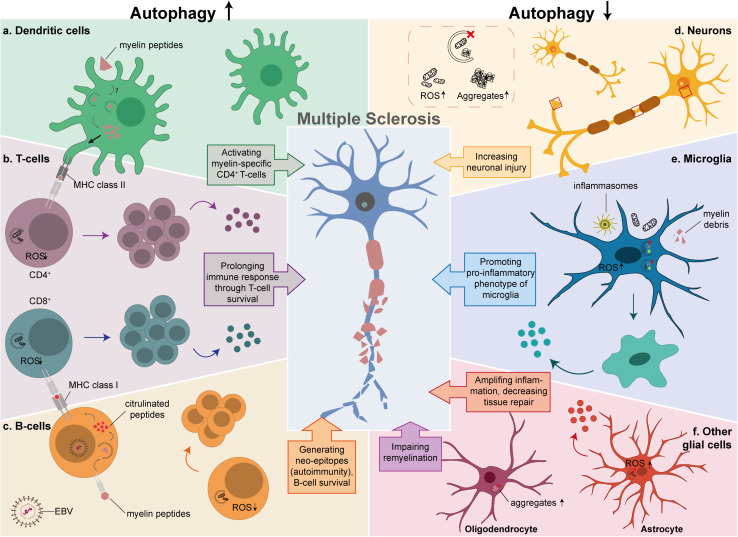
The possible link between autophagy and MS in different cell types. Autophagy is critical in the development and function of cells that play an important role in MS pathology. The left side of the diagram shows the effects of an enhancement of autophagy in T-cells, B-cells and DCs, while the right side depicts the effects of autophagy downregulation in neurons, microglia and other glial cells. **(a)** Increased myelin processing and antigen presentation to CD4+ autoreactive T-cells in DCs by either autophagy or LAP. **(b)** Prolonged survival of activated CD4+ and CD8+ T-cells due to low levels of ROS, resulting in proliferation and secretion of pro-inflammatory cytokines. **(c)** Productive processing of antigens in EBV-infected B-cells, that results in citrullinated peptides that are presented as neo-epitopes to CD8+ T-cells, and prolonged survival of B-cells by degrading damaged mitochondria. **(d)** Defective autophagy in neurons results in increased ROS levels and aggregate formation. **(e)** Insufficient clearance of damaged mitochondria, inflammasomes, and myelin debris in microglia, promotes a pro-inflammatory phenotype caused by autophagy or LAP. **(f)** Decreased tissue repair and secretion of pro-inflammatory cytokines by astrocytes (red), aggravates the inflammatory response and impaired remyelination by OLs (purple).

DCs phagocytose antigens and after their processing, the resulting peptides are presented on MHC class I and II molecules on the cell surface to activate CD8^+^ and CD4^+^ T-cells, respectively. During immune activation, autophagy is involved in host protection by delivering cytoplasmic antigens to lysosomes for subsequent presentation on MHC class II (Paludan et al., 2005; Bhattacharya et al., 2014; Yang et al., 2015; Schmid et al., 2007). In addition, extracellular compounds are degraded by LC3-associated phagocytosis (LAP), which depends on several ATG proteins (Lai and Devenish, 2012). This suggests that the ATG machinery might be involved in the myelin peptide presentation on MHC class II molecules and subsequently activation of CD4^+^ autoreactive T-cells (Figure 2a). Studies supporting this hypothesis showed that DCs lacking *Atg5* or *Atg7* reduced the incidence and severity of EAE (Bhattacharya et al., 2014; Keller et al., 2017; Hassanpour et al., 2020). The absence of ATG proteins in DCs caused a reduction of myelin peptide presentation and less activated CD4^+^ T-cells during EAE, however, it did not affect the levels of CD8^+^ T-cells (Bhattacharya et al., 2014; Keller et al., 2017; Hassanpour et al., 2020). Interestingly, autophagy-deficient DCs completely inhibited the development of EAE via adoptive transfer of primed encephalitogenic T-cells (Keller et al., 2017), suggesting that ATG proteins are important for the activation of primed myelin-specific CD4^+^ T-cells. Moreover, deletion of *ATG* genes in DCs did not affect other functions of DCs (Lee et al., 2010; Bhattacharya et al., 2014; Keller et al., 2017), indicating their specific importance for antigen presentation. Pharmacological inhibition of autophagy with chloroquine before EAE onset delayed disease progression and reduced EAE severity when administered during EAE development (Bhattacharya et al., 2014). However, this approach is not specific for autophagy and also affects LAP as well as other processes relying on lysosomal proteolytic activity. Therefore, further investigation is necessary to reveal whether autophagy is involved in antigen presentation of myelin-derived peptides in DCs or whether this is regulated by ATG protein-dependent phagocytic processes.

Altogether, DCs have both protective and pathological roles in MS, and autophagy could be important for the CD4^+^ T-cell-mediated autoimmune responses, thereby contributing to the pathological traits of DCs in MS.

### T-Cells

T-cells originate from bone marrow-derived hematopoietic stem cells. Lymphoid precursor cells migrate via the blood to the thymus where they develop into mature T-lymphocytes (Jia and He, 2011; Parekh et al., 2013; Bronietzki et al., 2015). T-cells are part of the adaptive immune system and are important players in both the development and modulation of inflammation. It is generally accepted that autoreactive T-cells against myelin in the CNS are key contributors to MS pathology (Group Nature Publishing, 2001; Glass et al., 2010; Liang and Le, 2015). The current notion is that T-cells are activated in the periphery by APCs, in particular by DCs, and differentiate into autoreactive T-cells. These autoreactive T-cells enter the CNS by damaging the blood-brain barrier, and in the CNS, they get reactivated and amplified (Group Nature Publishing, 2001; Glass et al., 2010; Chihara, 2018), and attack myelin sheaths of axons, resulting in denuded axons and ultimately in neuronal loss (Group Nature Publishing, 2001; Glass et al., 2010). Although, MS is thought to be a CD4^+^ T-cell-mediated autoimmune disease, an increasing number of studies has reported a role of CD8^+^ T-cells in the initial relapse phase of MS since the frequency of CD8+ T-cells appearance in lesions was increased (Friese and Fugger, 2009; Salou et al., 2015). Several studies also highlighted the importance of T-cells in MS pathology; they showed that the balance between CD4^+^ T-cells, CD8^+^ T-cells, and Tregs is disturbed (Fletcher et al., 2010; Chihara, 2018). This might be due to higher levels of autoreactive T-cells that showed increased proliferation and prolonged survival in MS patients (Sawcer et al., 2011; Igci et al., 2016).

During the past decades, autophagy has been implicated in various biological processes of T-cells, such as maintenance of T-cell homeostasis, differentiation, and activation (Li et al., 2006; Pua and He, 2007; Pua et al., 2007, 2009; Botbol et al., 2016; Paunovic et al., 2018; Macian, 2019). The expression levels of the *ATG5* gene in T-cells from MS patients are increased in blood and brain sections (Alirezaei et al., 2009; Yang et al., 2015), indicating a possible involvement of autophagy in the activation of autoreactive T-cells. Consistently, autophagosomes were only detected in CD4^+^ T-cells after T-cell receptor activation, and not in resting, naïve cells (Li et al., 2006; Hubbard et al., 2010; Macian, 2019). Mice experiments with either *Atg5-* or *Atg7-*deficient CD4^+^ and CD8^+^ T-cells showed indeed multiple defects, including reduced survival and a defect in T-cell proliferation in response to antigen stimulation (Pua et al., 2007; Alirezaei et al., 2009; Keller and Lünemann, 2017; Paunovic et al., 2018; Yin et al., 2018). *Beclin1*-deficient CD4^+^ T-cells prevented EAE development in mice, and T-cells were absent in the CNS (Alirezaei et al., 2009; Kovacs et al., 2012; Yin et al., 2018).

It has also been suggested that autophagy regulates cell death in activated T-cells (Kovacs et al., 2012). *Beclin1*-deficient CD4^+^ T-cells are more susceptible to apoptotic stimuli since they accumulate cell death-related proteins, such as procaspase-3, procaspase-8, and BCL2-interacting mediator (BIM). In particular, cell death-related proteins have been found in autophagosomes, and these proteins accumulated in autophagy-deficient T-cells (Li et al., 2006; Pua and He, 2007; Trapp and Nave, 2008; Pua et al., 2009; Kovacs et al., 2012; Salminen et al., 2013). This suggests a pro-survival function of autophagy in activated T-cells through the turnover of cell death-related proteins, which then will prolong their survival and consequent rapid amplification in the CNS that will initiate a persisting immune response (Figure 2b; Botbol et al., 2016). In addition, other reports have revealed that organelle turnover in T-cells critically depends on autophagy. Specifically, ER and dysfunctional mitochondria accumulate in T-cells when autophagy is blocked, which in turn leads to an increase in ROS levels and consequent cell death (Pua et al., 2009; Hubbard et al., 2010; Jia and He, 2011; Kovacs et al., 2012; Macian, 2019). An important finding is that subtypes of T-cells such as Th17 and Th1 are not equally susceptible to cell death after *Beclin1*-deletion (Kovacs et al., 2012), which might be due to the importance of autophagy in cell survival in different subsets of T-cells, or other roles of Beclin1 outside the context of autophagy.

These findings show that enhanced autophagy promotes T-lymphocyte survival and proliferation, thereby positively contributing to MS pathogenesis.

### B-Cells

B-cells play an important role in immune processes by generating antibodies that are directed to pathogens (Lehmann Horn et al., 2013; Li et al., 2018). Moreover, B-cells are recognized as APCs and thereby contribute to the regulation of immune processes (Hirotani et al., 2010; von Büdingen et al., 2015; Arneth, 2019). The crucial role of B-cells in MS pathology became clear when depletion of B-cells in MS patients with anti-CD20 antibodies led to the suppression of an inflammatory response, reducing the formation of new lesions and disease progression (Hauser et al., 2008; Gelfand et al., 2017; Mulero et al., 2018; Sospedra, 2018; Arneth, 2019). Similar to DCs and T-cells, B-cells consist of different subpopulations. B-cells in MS patients show increased secretion of pro-inflammatory cytokines (Bar-Or et al., 2010) and a deficiency in IL-10 production (Duddy et al., 2007), suggesting a perturbed balance between pro-inflammatory and regulatory B-cells, respectively. It is not fully understood how these B-cells contribute to MS pathology.

One environmental risk factor that has been linked to MS is the Epstein–Barr virus (EBV) (Sospedra, 2018). EBV infects B-cells, which in turn cross-present autoantigens that can activate T-cells against myelin (Bar-Or et al., 2020). The link between EBV and MS development is quite strong since nearly all MS patients had a past EBV infection (Ascherio and Munger, 2010; Guan et al., 2019). It appears that EBV infection during adolescence is a prerequisite to develop MS, although not sufficient on its own (Ascherio and Munger, 2010; Guan et al., 2019; Bar-Or et al., 2020). B-cells from MS patients show an increased expression of APC-related markers (Sospedra, 2018; Guan et al., 2019) and experiments in EAE mice uncovered that EBV upregulates antigen cross-presentation of infected B-cells to CD8^+^ T-cells (Dunham et al., 2017; Jakimovski et al., 2017). These results indicate that EBV influences the antigen presentation of B-cells. This notion is also supported by EAE animal experiments, where uninfected B-cells prevented autoimmunity by degrading self-antigens, while these antigens, which are generated by the productive processing of myelin oligodendrocyte glycoprotein (MOG), are presented to autoreactive T-cells in EBV infected B-cells, thereby inducing an immune activity (Thorley-Lawson and Mann, 1985; Livingston et al., 1997; Jagessar et al., 2016; Dunham et al., 2017; Jakimovski et al., 2017; Morandi et al., 2017; Guan et al., 2019).

It has been suggested that the productive processing of antigens results from the citrullination of peptides, and this is enhanced by an EBV infection (’t Hart et al., 2016; Morandi et al., 2017; Bar-Or et al., 2020). Citrullination is a posttranslational modification that converts arginine into citrulline, this conversion is relevant for antigen presentation because it generates neo-epitopes that can be recognized by the immune system (Guan et al., 2019). Autophagy is responsible for the generation and processing of citrullinated peptides (Ireland and Unanue, 2011; Münz, 2016; Morandi et al., 2017), resulting in neo-epitopes that could be recognized by T-cells and induce an autoimmune response (Alghamdi et al., 2019). An interesting finding has been that the processing of citrullinated peptides depends on autophagy induction in B-cells, whereas unmodified peptides are unaffected when autophagy was blocked in this cell type with 3-methyladenine (Ireland and Unanue, 2011). In particular, citrullination of the MOG peptide at Arg46 protected this peptide from degradation in EBV-infected B-cells. Interestingly, Arg46 in MOG is positioned within the LIR motif that is important for its selective targeting by autophagy (Birgisdottir et al., 2013; Morandi et al., 2017). These findings suggest a mechanistic link between EBV, autophagy, and autoimmunity. EBV-infected B-cells indeed display more autophagosomes, and MOG peptides are present inside these vesicles (Ireland and Unanue, 2011; Morandi et al., 2017). Moreover, pharmacological induction of autophagy with rapamycin further enhanced the protection of citrullinated MOG peptides from degradation (Camilli et al., 2016; Morandi et al., 2017), indicating that this pathway protects myelin peptides against destructive processing and consequently promotes their presentation to T-cells. Altogether, EBV infection in B-cells is responsible for inducing autophagy, which is important for altering antigens that can initiate autoimmunity against myelin in MS (Figure 2c).

In addition to the role in the generation and processing of citrullinated peptides in EBV-infected B-cells, autophagy is also important for B-cell survival, development, and activation (Rathmell, 2012; Puleston and Simon, 2014; Bhattacharya and Eissa, 2015), similarly to what happens in T-cells (see section “T-Cells”). Thus, like DCs and T-cells, autophagy activation in B-cells appears to contribute to the pathogenicity of MS rather than to its prevention.

### Neurons

Currently, axonal damage is considered part of a secondary phase of MS, which is caused by an initial inflammation in the periphery that is subsequently followed by demyelination in the CNS (Ferguson et al., 1997; Trapp et al., 1998; Tsunoda and Fujinami, 2002). This concept is known as the outside-in model. However, this model is debated questioning whether the axonal injury is exclusively caused by an immune response initiated in the periphery or directly from the neurons. Moreover, it cannot be excluded that neuronal loss is the primary phase of MS, which is then followed by a second phase characterized by demyelination and an inflammation response (Lovas et al., 2000; Bjartmar et al., 2001; Tsunoda and Fujinami, 2002). This scenario is known as the inside-out model. Infections in neurons can indeed induce neuronal damage, which leads to demyelination and neurodegeneration (Tsunoda et al., 2003), and these observations support the inside-out model. However, there are also examples from experiments with animal models of MS that showed evidence of axonal injury without any signs of demyelination (Ferguson et al., 1997; Trapp et al., 1998; Tsunoda et al., 2003). Thus, it is possible that in addition to demyelination, other triggers are involved in the induction of neuronal loss during MS (Tsunoda and Fujinami, 2002).

Neurons depend on autophagy for clearing misfolded or aggregated proteins and damaged organelles, and autophagy is continuously active at basal levels in neuronal cells under normal conditions (Hara et al., 2006; Plaza-Zabala et al., 2017; Feng et al., 2018; Stavoe and Holzbaur, 2019). Autophagy is active in each neuronal compartment, however, the axons and dendrites are the most metabolically demanding regions where autophagy is crucial (Stavoe and Holzbaur, 2019). It is known that basal autophagy in neurons is essential for protein quality control, pruning, development, and neuronal survival (Hara et al., 2006; Komatsu et al., 2006; Wong and Cuervo, 2010; Feng et al., 2017; Plaza-Zabala et al., 2017; Stavoe and Holzbaur, 2019). Defects in neuronal autophagy results in aggregate formation and neuronal damage, which ultimately leads to neuronal death (Hara et al., 2006; Komatsu et al., 2006, 2007; Liang and Le, 2015; Feng et al., 2017; Stavoe and Holzbaur, 2019; Figure 2d). Defective autophagy has been observed in the spinal cords of EAE mice, and pharmacological induction of autophagy with rapamycin reduced demyelination, inflammation, and neuronal loss (Feng et al., 2017, 2018). In contrast, inhibition of autophagy non-specifically with 3-methyladenine, resulted in higher neuronal apoptosis in EAE mice (Feng et al., 2017), suggesting that autophagy dysfunction could be associated with EAE-induced neuronal loss. Another study showed that LC3 protein expression levels in neurons were higher in control mice compared to EAE mice (Feng et al., 2018), however, this could indicate that autophagy is either reduced or enhanced in neurons during EAE development. Future research has to reveal whether neuronal autophagy contributes to the neurobiological and neuropathological features of MS.

### Microglia

Microglia are the tissue-resident macrophages of the CNS and they form the first line of defense in the CNS (Schulz et al., 2012; Kierdorf et al., 2013; Luo et al., 2017). Microglia get activated upon tissue injury or a stimulus via a variety of cell surface receptors (Augusto-Oliveira et al., 2019). Activated microglia are essential for inflammatory responses in the CNS (Ponomarev et al., 2005; Luo et al., 2017), where they are involved in phagocytosis, antigen presentation, and cytokine production (Benveniste, 1997). Microglia activation can result in either neurotoxic or neuroprotective effects, depending on the stimulus (Orihuela et al., 2016).

Activated microglia are present in CNS lesions of MS patients and animal models, and are found to be an important source of ROS and nitric oxide (NO) radicals (Gray et al., 2008; Zeis et al., 2009). Interestingly, genes identified to be associated with MS susceptibility are enriched in microglia compared to other CNS cell types (Patsopoulos et al., 2019; Guerrero and Sicotte, 2020), placing these cells in the spotlight of the disease. Nowadays, microglia are recognized as one of the key players in MS pathophysiology. However, the role of microglia in MS is complex and controversial. Microglia are heterogeneous cells that can adopt a range of different phenotypes, with different functions, in response to different stimuli (Durafourt et al., 2012; Melief et al., 2012, 2013; Boche et al., 2013; Giunti et al., 2014). A few studies have shown that activated microglia participate in both the inflammation state and demyelination, by secreting pro-inflammatory cytokines (Prineas et al., 2001; Lassmann et al., 2007; Luo et al., 2017). Microglia-deficient EAE mice are protected against gray and white matter damage (Heppner et al., 2005), and EAE severity is reduced (Bogie et al., 2014). Inhibition of microglial activation in EAE mice also resulted in a reduction of demyelination and preserved mature oligodendrocytes (OLs) (further discussed in the next section) (Nissen et al., 2018). Additionally, microglia-deficient mice showed a reduction in myelin debris clearance, resulting in impaired remyelination (Lampron et al., 2015). Microglia promote remyelination by secreting anti-inflammatory cytokines, phagocytosing myelin debris (Prineas et al., 2001; De Groot et al., 2001; Lassmann et al., 2007; Kierdorf et al., 2013; Guerrero and Sicotte, 2020), and enhancing OLs proliferation and differentiation (Li et al., 2005; Voß et al., 2012; Miron et al., 2013; Bogie et al., 2014; Lloyd et al., 2017). Taken together, microglia are involved in different phases of MS, in which they play either a pathological or a protective role.

It has been postulated that autophagy is involved in microglia-mediated neuroinflammation since there is evidence that links autophagy to the regulation of microglial inflammation (Plaza-Zabala et al., 2017). Autophagy induction in pre-stimulated microglial cells with an inflammatory stimulus, tumor necrosis factor α (TNF-α) or lipopolysaccharide (LPS), promotes microglia toward an anti-inflammatory phenotype and suppresses pro-inflammatory genes (Shao et al., 2014; Su et al., 2016; Bussi et al., 2017; He et al., 2018; Jin et al., 2018; Hassanpour et al., 2020). Conversely, autophagy inhibition leads to opposite results, regardless of the presence of an inflammatory stimulus (Shao et al., 2014; Su et al., 2016; Bussi et al., 2017; He et al., 2018; Jin et al., 2018; Hassanpour et al., 2020). Moreover, *Atg5* knockdown in microglia, enhances neurotoxicity in microglia-neuron co-cultures (Bussi et al., 2017; He et al., 2018; Jin et al., 2018), while autophagy induction by activating cannabinoid receptor 2 prevents inflammasome activation in both EAE mice (Shao et al., 2014) and microglia cultures (Shao et al., 2014; Su et al., 2016). Together, these observations indicate that autophagy is a key process in microglia as it balances their pro- and anti-inflammatory responses (Figure 2e).

Besides the involvement of microglial autophagy in inflammatory responses, ATG proteins are also involved in the phagocytosis and elimination of myelin debris (Sanjuan et al., 2007), which indicates the possible involvement of LAP. As a result, defective ATG machinery in microglia could lead to an inefficient clearing of myelin debris, which in turn will cause impairment in remyelination and enhanced neuroinflammation in neurodegenerative diseases (Sanjuan et al., 2007; Meikle et al., 2008; Rangaraju et al., 2010). Altogether, these observations emphasize the importance of microglial autophagy and ATG proteins in general, in MS etiology since they negatively modulate the underlying inflammatory response and promote remyelination.

Microglia are also involved in synaptic pruning during development. However, they also play a role in the synaptic loss seen in neurodegenerative conditions, such as MS (Ramaglia et al., 2012). This action requires complement C3 that localizes to synapses which are then recognized by complement receptors expressed by microglia (Ramaglia et al., 2012; Werneburg et al., 2020). Besides their importance in synaptic pruning, these complement molecules are also involved in microglia priming which leads to an exaggerated response to a potentially minor secondary stimulus which is also connected to MS. Interestingly, besides neuronal autophagy, autophagy in microglia has also shown to control synaptic pruning (Druart and Le Magueresse, 2019; Lieberman et al., 2019). Mice that were Atg7-deficient specifically in microglia showed increased spine density (Kim et al., 2017; Lieberman et al., 2019). One of the hypotheses is that autophagy in microglia is important for degrading the phagocytosed components by microglial cells and this could be performed by LAP, which overlaps extensively with the conventional autophagy pathway (Lieberman et al., 2019). Both neuronal and microglial autophagy are involved in synaptic development and dysfunction of this process might also be involved in synaptic loss seen in MS. Further investigation is required to reveal whether inflammation is the main cause of the pathogenesis or whether dysfunction in autophagy causes both inflammation and neurodegeneration.

### Oligodendrocytes

In addition to microglia, activation of OLs are also important in neuroinflammation and are involved in the development of MS (Glass et al., 2010; Liang and Le, 2015).

Oligodendrocytes differentiate from oligodendrocyte precursor cells (OPCs) and are important for the myelination of axons in the CNS (Nave and Werner, 2014), where the extensive loss of OLs has been observed in MS lesions (Wolswijk, 2000). In MS, several processes result in the injury of both OLs and OPCs, leading to demyelination and inefficient remyelination, respectively (Chang et al., 2000; Wolswijk, 2002). Autophagy is important for the survival and differentiation of OLs, and it influences their myelinating ability (Bankston et al., 2019). The enhancement of autophagy increases the thickness of the myelin sheaths as well as the numbers of myelinated axons (Smith et al., 2013). Moreover, autophagy-deficient OLs showed a reduction in the number of myelinated axons and decreased thickness of the myelin (Bankston et al., 2019). It has been suggested that a key function of autophagy in OLs is to prevent aggregation of myelin components, allowing OLs to continue with protein and lipid synthesis to form compact myelin sheaths (Figure 2f; Smith et al., 2013). Dysfunction of autophagy in OLs might also play a role in the field of myelin plasticity, where it may be involved in cytoplasm decompaction and decreased numbers of myelin wraps due to lower levels of OLs that ultimately leads to demyelination in MS (Hill et al., 2018; Belgrad et al., 2020). However, the exact role of autophagy in OLs and whether the disrupted OLs protein homeostasis in MS is caused by an autophagy impairment, remain to be clarified.

### Astrocytes

Another important cell type in MS are astrocytes, which supports and regulates the communication between neurons and maintains the blood-brain barrier. They also participate in CNS damage repair by secreting growth factors and extracellular matrix proteins (Joe et al., 2018). Several studies have shown that astrocytes have multiple functions in the formation of MS lesions, where they can be activated during the inflammatory process and release inflammatory mediators that aggravate brain lesions (Cotrina and Nedergaard, 2002; Cornejo et al., 2018; Ponath et al., 2018; Cohen and Torres, 2019; Cressatti et al., 2019). They can also recruit peripheral immune cells to the inflammation site of the CNS (Rezai-Zadeh et al., 2009; Chompre et al., 2013; Clarke et al., 2018). Astrocytes are also involved in the repair of lesions, restricting the inflammatory damage (Sofroniew and Vinters, 2010; Cho et al., 2014). Genetic astrocyte ablation in MS mouse models aggravated tissue damage and clinical impairment by both preventing the recruitment of microglia to clear myelin debris and reducing the proliferation of OPCs (Brambilla et al., 2009, 2005; Skripuletz et al., 2013). These events result in impaired remyelination and shows the importance of astrocytes in promoting tissue repair.

Autophagy in astrocytes is important for their differentiation and maturation (Wang et al., 2014; Wang and Xu, 2020), and it is implicated in the role of astrocytes in several neurodegenerative diseases besides MS (Wang and Xu, 2020), including PD and AD. In particular, autophagy in astrocytes is important in regulating mitochondria dynamics and preserving mitochondrial network organization during inflammation. Consequently, impairment of this process results in the generation of ROS, which in turn amplifies the pro-inflammatory response and ultimately leads to the cell death of astrocytes (Lee et al., 2009; Motori et al., 2013). Moreover, autophagy in astrocytes has also been linked to neuronal survival since its inhibition with either rapamycin or transduction with small interfering RNA against *Atg5* induces neuronal death (Figure 2f; Malta et al., 2012; Liu et al., 2018). Together, these results underline the important role of autophagy in astrocytes to maintain homeostasis in an inflammatory environment, which contributes to neuronal survival. Whether autophagy is dysregulated in astrocytes during MS needs to be further investigated.

## Discussion

Defects in autophagy contribute to MS etiology. Autophagy, however, acts as a two-edged sword during MS, having both protective and detrimental effects that are cell type-dependent. As highlighted in this review, autophagy enhancement in cell types like DCs, T-cells, and B-cells, is participating to the initiation of neuroinflammation seen in MS. Inhibition of autophagy in these cells could be a potential therapeutic target. Yet, autophagy also appears to be protective against the detrimental effects of the immune system in neurons and glial cells, where it prevents both aggregate and ROS formation, modulates the inflammatory response, and promotes remyelination. To connect the role of autophagy in MS to one of the paradigms in MS etiopathogenesis (“inside-out” or “outside-in”) based on the current knowledge is difficult. Autophagy is involved in both inflammation and neurodegeneration processes that are seen in MS. The findings that link autophagy to the pathology of DCs, T-cells, and B-cells, could be considered as an “outside-in” event. However, the functional role of autophagy in neurons which is affected in MS and clearance of myelin debris by glial cells could be considered as an “inside-out” event. How the autophagy process is affected in these different cell types is an important question that needs to be answered in order to have a significant input in the ongoing debate whether MS is an “inside-out” or “outside-in” event.

Thus, the available data suggest that autophagy plays an important role in the regulation of the immune response under normal conditions and in preventing the development of an autoimmune response. This raises the possibility that modulating the autophagy process in a cell type-specific manner may limit inflammatory CNS damage and demyelination over the course of MS, which in turn would protect against neuronal death. It might be possible that the involvement of ATG genes in the phagocytosis of extracellular myelin debris and other components by DCs and microglia is rather due to LAP. However, autophagy and LAP share numerous ATG proteins, and therefore it is difficult to distinguish between the two. One known difference between autophagy and LAP is the requirement of ULK kinase complex in autophagy and not in LAP (Lai and Devenish, 2012). Additionally, ultrastructural observations of the phagosome membrane might reveal the contribution and importance of these processes in MS pathology.

In the optic of future therapies, it will be important to elucidate whether autophagy modulation is beneficial in both relapsing-remitting and progressive MS patients. However, autophagy might be more therapeutically beneficial for relapsing-remitting patients since this phase includes active inflammatory demyelinating lesions, while this phenomenology is absent in chronic progressive lesions (Dutta and Trapp, 2014).

Pharmacological interventions targeting autophagy in specific cell types might help to restore the balance of the immune system, which is a promising avenue for the treatment of autoimmune disorders. Most of the current pharmacological modulators of autophagy act on signaling cascades that regulate this process (Figure 1), rather than specifically target autophagy itself. This could result in off-target effects, which could be avoided by giving the treatment in cycles of brief periods. On the other hand, more direct biochemical approaches to modulate autophagy such as spermidine (Morselli et al., 2011) and TAT-beclin (Shoji-Kawata et al., 2013), are promising for the treatment of MS as they are also less invasive. Moreover, caloric restriction or exercise enhances autophagy and therefore might be effective as a treatment for MS (Choi et al., 2016).

Based on the current knowledge about the involvement of autophagy in different cell types during MS, T-cells and microglia are promising targets for cell type-specific delivery of autophagy modulators (Zhang F. et al., 2016; Schmid et al., 2017; Wang et al., 2019). In this context, nanoparticles that specifically bind to particular T-cell subsets have been designed (Schmid et al., 2017), and inhibiting autophagy in CD4^+^ and CD8^+^ autoreactive T-cells could prevent the initial activation of the immune response seen in MS. Since prolonged inhibition of autophagy in T-cells might negatively affect T-cell homeostasis, transient therapy is desirable. In addition, autophagy inducers in nanoparticles that are specifically targeted to microglia and macrophages (Wang et al., 2019) could selectively promote both anti-inflammatory responses and dampening of the pro-inflammatory effects, which will ultimately result in beneficial effects on the inflammation resolution, clearing of myelin debris, and remyelination. However, additional research is needed to investigate whether a nanoparticle or any other approach to either block or stimulate autophagy in a cell type-specific manner can delay MS progression. Nonetheless, autophagy is an attractive and promising target for the development of new treatments for MS and future studies investigating the precise role of this pathway in the different cell types during the course of this severe disease will be key to appropriately intervene therapeutically.

## Author Contributions

CM wrote the manuscript. MM, FR, and BE edited the manuscript. All authors contributed to the article and approved the submitted version.

## Conflict of Interest

The authors declare that the research was conducted in the absence of any commercial or financial relationships that could be construed as a potential conflict of interest.

## References

[B1] Albert M. Barrantes-Freer A. Lohrberg M. Antel J. P. Prineas J. W. Palkovits M. (2017). Synaptic pathology in the cerebellar dentate nucleus in chronic multiple sclerosis. *Brain Pathol.* 27 737–747. 10.1111/bpa.12450 27706868 PMC8028945

[B2] Alirezaei M. Fox H. S. Flynn C. T. Moore C. S. Hebb A. L. Frausto R. F. (2009). Elevated ATG5 expression in autoimmune demyelination and multiple sclerosis. *Autophagy* 5 152–158. 10.4161/auto.5.2.7348 19066443 PMC2779564

[B3] Alghamdi M. Alasmari D. Assiri A. Mattar E. Aljaddawi A. A. Alattas S. G. (2019). An overview of the intrinsic role of citrullination in autoimmune disorders. *J. Immunol. Res.* 2019:7592851. 10.1155/2019/7592851 31886309 PMC6899306

[B4] Arneth B. M. (2019). Impact of B cells to the pathophysiology of multiple sclerosis. *J. Neuroinflamm.* 16:128. 10.1186/s12974-019-1517-1 31238945 PMC6593488

[B5] Ascherio A. Munger K. L. (2010). Epstein-Barr virus infection and multiple sclerosis: a review. *J. Neuroimmune Pharmacol.* 5 271–277. 10.1007/s11481-010-9201-3 20369303

[B6] Augusto-Oliveira M. Arrifano G. P. Lopes-Araújo A. Santos-Sacramento L. Takeda P. Y. Anthony D. C. (2019). What do microglia really do in healthy adult brain? *Cells* 8:1293. 10.3390/cells8101293 31652490 PMC6829860

[B7] Bankston A. N. Forston M. D. Howard R. M. Andres K. R. Smith A. E. Ohri S. S. (2019). Autophagy is essential for oligodendrocyte differentiation, survival, and proper myelination. *Glia* 67 1745–1759. 10.1002/glia.23646 31162728

[B8] Bar-Or A. Fawaz L. Fan B. Darlington P. J. Rieger A. Ghorayeb C. (2010). Abnormal B-cell cytokine responses a trigger of T-cell-mediated disease in MS? *Ann. Neurol.* 67 452–461. 10.1002/ana.21939 20437580

[B9] Bar-Or A. Pender M. P. Khanna R. Steinman L. Hartung H. P. Maniar T. (2020). Epstein–Barr virus in multiple sclerosis: theory and emerging immunotherapies. *Trends Mol. Med.* 26 296–310. 10.1016/j.molmed.2019.11.003 31862243 PMC7106557

[B10] Beau I. Mehrpour M. Codogno P. (2011). Autophagosomes and human diseases. *Int. J. Biochem. Cell Biol.* 43 460–464. 10.1016/j.biocel.2011.01.006 21256243

[B11] Belgrad J. De Pace R. Fields R. D. (2020). Autophagy in myelinating glia. *J. Neurosci.* 40 256–266. 10.1523/JNEUROSCI.1066-19.2019 31744863 PMC6948934

[B12] Benveniste E. N. (1997). Cytokines: influence on glial cell gene expression and function. *Chem. Immunol.* 69 31–75. 10.1159/000058653 9353961

[B13] Bhattacharya A. Eissa N. T. (2015). Autophagy as a stress response pathway in the immune system. *Int. Rev. Immunol.* 34 382–402. 10.3109/08830185.2014.999156 25699578

[B14] Bhattacharya A. Parillon X. Zeng S. Han S. Eissa N. T. (2014). Deficiency of autophagy in dendritic cells protects against experimental autoimmune encephalomyelitis. *J. Biol. Chem.* 289 26525–26532. 10.1074/jbc.M114.575860 25077962 PMC4176242

[B15] Birgisdottir ÅB. Lamark T. Johansen T. (2013). The LIR motif - crucial for selective autophagy. *J. Cell Sci.* 126 3237–3247. 10.1242/jcs.126128 23908376

[B16] Bjartmar C. Kinkel R. P. Kidd G. Rudick R. A. Trapp B. D. (2001). Axonal loss in normal-appearing white matter in a patient with acute MS. *Neurology* 57 1248–1252. 10.1212/WNL.57.7.1248 11591844

[B17] Boche D. Perry V. H. Nicoll J. A. (2013). Review: activation patterns of microglia and their identification in the human brain. *Neuropathol. Appl. Neurobiol.* 39 3–18. 10.1111/nan.12011 23252647

[B18] Bogie J. F. Stinissen P. Hendriks J. J. (2014). Macrophage subsets and microglia in multiple sclerosis. *Acta Neuropathol.* 128 191–213. 10.1007/s00401-014-1310-2 24952885

[B19] Botbol Y. Guerrero-Ros I. Macian F. (2016). Key roles of autophagy in regulating T-cell function. *Eur. J. Immunol.* 46 1326–1334. 10.1002/eji.201545955 27151577 PMC5227655

[B20] Boyao Y. Mengjiao S. Caicai B. Xiaoling L. Zhenxing L. Manxia W. (2019). Dynamic expression of autophagy-related factors in autoimmune encephalomyelitis and exploration of curcumin therapy. *J. Neuroimmunol.* 337:577067. 10.1016/j.jneuroim.2019.577067 31629984

[B21] Brambilla R. Bracchi-Ricard V. Hu W. H. Frydel B. Bramwell A. Karmally S. (2005). Inhibition of astroglial nuclear factor K B reduces inflammation and improves functional recovery after spinal cord injury. *J. Exp. Med.* 202 145–156. 10.1084/jem.20041918 15998793 PMC2212896

[B22] Brambilla R. Persaud T. Hu X. Karmally S. Shestopalov V. I. Dvoriantchikova G. (2009). Transgenic inhibition of astroglial NF-K B improves functional outcome in experimental autoimmune encephalomyelitis by suppressing chronic central nervous system inflammation. *J. Immunol.* 182 2628–2640. 10.4049/jimmunol.0802954 19234157 PMC4291126

[B23] Bronietzki A. W. Schuster M. Schmitz I. (2015). Autophagy in T-cell development, activation and differentiation. *Immunol. Cell Biol.* 93 25–34. 10.1038/icb.2014.81 25287445

[B24] Bussi C. Ramos J. Arroyo D. Gaviglio E. Gallea J. Wang J. (2017). Autophagy down regulates pro-inflammatory mediators in BV2 microglial cells and rescues both LPS and alpha-synuclein induced neuronal cell death. *Sci. Rep.* 7:43153. 10.1038/srep43153 28256519 PMC5335665

[B25] Camilli G. Cassotta A. Battella S. Palmieri G. Santoni A. Paladini F. (2016). Regulation and trafficking of the HLA-E molecules during monocyte-macrophage differentiation. *J. Leukoc. Biol.* 99 121–130. 10.1189/jlb.1a0415-172r 26310830

[B26] Chang A. Nishiyama A. Peterson J. Prineas J. Trapp B. D. (2000). NG2-positive oligodendrocyte progenitor cells in adult human brain and multiple sclerosis lesions. *J. Neurosci.* 20 6404–6412. 10.1523/JNEUROSCI.20-17-06404.2000 10964946 PMC6772992

[B27] Chen Y. McMillan-Ward E. Kong J. Israels S. J. Gibson S. B. (2008). Oxidative stress induces autophagic cell death independent of apoptosis in transformed and cancer cells. *Cell Death Differ.* 15 171–182. 10.1038/sj.cdd.4402233 17917680

[B28] Chihara N. (2018). Dysregulated T cells in multiple sclerosis. *Clin. Exp. Neuroimmunol.* 9 20–29. 10.1111/cen3.12438

[B29] Cho M. H. Cho K. Kang H. J. Jeon E. Y. Kim H. S. Kwon H. J. (2014). Autophagy in microglia degrades extracellular β-amyloid fibrils and regulates the NLRP3 inflammasome. *Autophagy* 10 1761–1775. 10.4161/auto.29647 25126727 PMC4198361

[B30] Choi A. M. Ryter S. W. Levine B. (2013). Mechanisms of disease: autophagy in human health and disease. *N. Engl. J. Med.* 368 651–662. 10.1056/NEJMra1205406 23406030

[B31] Choi I. Y. Piccio L. Childress P. Bollman B. Ghosh A. Brandhorst S. (2016). A diet mimicking fasting promotes regeneration and reduces autoimmunity and multiple sclerosis symptoms. *Cell Rep.* 15 2136–2146. 10.1016/j.celrep.2016.05.009 27239035 PMC4899145

[B32] Chompre G. Cruz E. Maldonado L. Rivera-Amill V. Porter J. T. Noel R. J. Jr. (2013). Astrocytic expression of HIV-1 Nef impairs spatial and recognition memory. *Neurobiol. Dis.* 49 128–136. 10.1016/j.nbd.2012.08.007 22926191 PMC3530662

[B33] Clarke A. J. Ellinghaus U. Cortini A. Stranks A. Simon A. K. Botto M. (2015). Autophagy is activated in systemic lupus erythematosus and required for plasmablast development. *Ann. Rheum. Dis.* 74 912–920. 10.1136/annrheumdis-2013-204343 24419333 PMC4152192

[B34] Clarke L. E. Liddelow S. A. Chakraborty C. Münch A. E. Heiman M. Barres B. A. (2018). Normal aging induces A1-like astrocyte reactivity. *Proc. Natl. Acad. Sci. U.S.A.* 115 E1896–E1905. 10.1073/pnas.1800165115 29437957 PMC5828643

[B35] Cohen J. Torres C. (2019). Astrocyte senescence: evidence and significance. *Aging Cell* 18:e12937. 10.1111/acel.12937 30815970 PMC6516680

[B36] Conway K. L. Kuballa P. Khor B. Zhang M. Shi H. N. Virgin H. W. (2013). ATG5 regulates plasma cell differentiation. *Autophagy* 9 528–537. 10.4161/auto.23484 23327930 PMC3627668

[B37] Cornejo F. Vruwink M. Metz C. Muñoz P. Salgado N. Poblete J. (2018). Scavenger receptor-A deficiency impairs immune response of microglia and astrocytes potentiating Alzheimer’s disease pathophysiology. *Brain Behav. Immun.* 69 336–350. 10.1016/j.bbi.2017.12.007 29246456

[B38] Cotrina M. L. Nedergaard M. (2002). Astrocytes in the aging brain. *J. Neurosci. Res.* 67 1–10. 10.1002/jnr.10121 11754075

[B39] Cressatti M. Song W. Turk A. Z. Garabed L. R. Benchaya J. A. Galindez C. (2019). Glial HMOX1 expression promotes central and peripheral α-synuclein dysregulation and pathogenicity in parkinsonian mice. *Glia* 67 1730–1744. 10.1002/glia.23645 31180611

[B40] Cuervo A. M. (2010). Chaperone-mediated autophagy: selectivity pays off. *Trends Endocrinol. Metab.* 21 142–150. 10.1016/j.tem.2009.10.003 19857975 PMC2831144

[B41] De Groot C. J. A. Bergers E. Kamphorst W. Ravid R. Polman C. H. Barkhof F. (2001). Post-mortem MRI-guided sampling of multiple sclerosis brain lesions: increased yield of active demyelinating and (p)reactive lesions. *Brain* 124 1635–1645. 10.1093/brain/124.8.1635 11459754

[B42] Deretic V. Saitoh T. Akira S. (2013). Autophagy in infection, inflammation and immunity. *Nat. Rev. Immunol.* 13 722–737. 10.1038/nri3532 24064518 PMC5340150

[B43] Djajadikerta A. Keshri S. Pavel M. Prestil R. Ryan L. Rubinsztein D. C. (2020). Autophagy induction as a therapeutic strategy for neurodegenerative diseases. *J. Mol. Biol.* 432 2799–2821. 10.1016/j.jmb.2019.12.035 31887286

[B44] Dobson R. Giovannoni G. (2019). Multiple Sclerosis–a review. *Eur. J. Neurol.* 26 27–40. 10.1111/ene.13819 30300457

[B45] Druart M. Le Magueresse C. (2019). Emerging roles of complement in psychiatric disorders. *Front. Psychiatry* 10:573. 10.3389/fpsyt.2019.00573 31496960 PMC6712161

[B46] Duddy M. Niino M. Adatia F. Hebert S. Freedman M. Atkins H. (2007). Distinct effector cytokine profiles of memory and naive human B cell subsets and implication in multiple sclerosis. *J. Immunol.* 178 6092–6099. 10.4049/jimmunol.178.10.6092 17475834

[B47] Dunham J. van Driel N. Eggen B. J. Paul C. ‘t Hart B. A. Laman J. D. (2017). Analysis of the Cross-Talk of Epstein–Barr virus-infected B cells with T cells in the marmoset. *Clin. Transl. Immunol.* 6:e127. 10.1038/cti.2017.1 28243437 PMC5311918

[B48] Durafourt B. A. Moore C. S. Zammit D. A. Johnson T. A. Zaguia F. Guiot M. C. (2012). Comparison of polarization properties of human adult microglia and blood-derived macrophages. *Glia* 60 717–727. 10.1002/glia.22298 22290798

[B49] Dutta R. Trapp B. D. (2014). Relapsing and progressive forms of multiple sclerosis: insights from pathology. *Curr. Opin. Neurol.* 27 271–278. 10.1097/WCO.0000000000000094 24722325 PMC4132635

[B50] Dyment D. A. Yee I. M. Ebers G. C. Sadovnick A. D. Canadian Collaborative Study Group (2006). Multiple sclerosis in stepsiblings: recurrence risk and ascertainment. *J. Neurol. Neurosurg. Psychiatry* 77 258–259. 10.1136/jnnp.2005.063008 16421134 PMC2077589

[B51] Esposito M. Ruffini F. Bellone M. Gagliani N. Battaglia M. Martino G. (2010). Rapamycin inhibits relapsing experimental autoimmune encephalomyelitis by both effector and regulatory T cells modulation. *J. Neuroimmunol.* 220 52–63. 10.1016/j.jneuroim.2010.01.001 20149931

[B52] Feng X. Hou H. Zou Y. Guo L. (2017). Defective autophagy is associated with neuronal injury in a mouse model of multiple sclerosis. *Bosn. J. Basic Med. Sci.* 17 95–103. 10.17305/bjbms.2017.1696 28086065 PMC5474114

[B53] Feng X. D. Yu S. S. Hou H. Q. Zou Y. L. Chen J. J. Guo L. (2018). Rapamycin reduces degeneration of neurons by inhibiting Akt/MTOR/P70S6K pathway and restoring autophagy in EAE mice. *Int. J. Clin. Exp. Med.* 11 3504–3513.

[B54] Feng Y. He D. Yao Z. Klionsky D. J. (2014). The machinery of macroautophagy. *Cell Res.* 24 24–41. 10.1038/cr.2013.168 24366339 PMC3879710

[B55] Ferguson B. Matyszak M. K. Esiri M. M. Perry V. H. (1997). Axonal damage in acute multiple sclerosis lesions. *Brain* 120 393–399. 10.1093/brain/120.3.393 9126051

[B56] Fletcher J. M. Lalor S. J. Sweeney C. M. Tubridy N. Mills K. H. G. (2010). T cells in multiple sclerosis and experimental autoimmune encephalomyelitis. *Clin. Exp. Immunol.* 162 1–11. 10.1111/j.1365-2249.2010.04143.x 20682002 PMC2990924

[B57] François A. Terro F. Quellard N. Fernandez B. Chassaing D. Janet T. (2014). Impairment of autophagy in the central nervous system during lipopolysaccharide-induced inflammatory stress in mice. *Mol. Brain* 7:56. 10.1186/s13041-014-0056-z 25169902 PMC4237961

[B58] Friese M. A. Fugger L. (2009). Pathogenic CD8 + T cells in multiple sclerosis. *Ann. Neurol.* 66 132–141. 10.1002/ana.21744 19743458

[B59] Fujikake N. Shin M. Shimizu S. (2018). Association between autophagy and neurodegenerative diseases. *Front. Neurosci.* 12:255. 10.3389/fnins.2018.00255 29872373 PMC5972210

[B60] Gelfand J. M. Cree B. A. C. Hauser S. L. (2017). Ocrelizumab and other CD20+ B-cell-depleting therapies in multiple sclerosis. *Neurotherapeutics* 14 835–841. 10.1007/s13311-017-0557-4 28695471 PMC5722762

[B61] Giunti D. Parodi B. Cordano C. Uccelli A. Kerlero de Rosbo N. (2014). Can we switch microglia’s phenotype to foster neuroprotection? Focus on multiple sclerosis. *Immunology* 141 328–339. 10.1111/imm.12177 24116890 PMC3930371

[B62] Glass C. K. Saijo K. Winner B. Marchetto M. C. Gage F. H. (2010). Mechanisms underlying inflammation in neurodegeneration. *Cell* 140 918–934. 10.1016/j.cell.2010.02.016 20303880 PMC2873093

[B63] Gray E. Thomas T. L. Betmouni S. Scolding N. Love S. (2008). Elevated myeloperoxidase activity in white matter in multiple sclerosis. *Neurosci. Lett.* 444 195–198. 10.1016/j.neulet.2008.08.035 18723077

[B64] Greter M. Heppner F. L. Lemos M. P. Odermatt B. M. Goebels N. Laufer T. (2005). Dendritic cells permit immune invasion of the CNS in an animal model of multiple sclerosis. *Nat. Med.* 11 328–334. 10.1038/nm1197 15735653

[B65] Group Nature Publishing (2001). Multiple sclerosis: a two-stage Disease. *Nat. Immunol.* 2 762–764. 10.1038/ni0901-762 11526378

[B66] Guan Y. Jakimovski D. Ramanathan M. Weinstock-Guttman B. Zivadinov R. (2019). The Role of Epstein-Barr virus in multiple sclerosis: from molecular pathophysiology to in vivo imaging. *Neural Regen. Res.* 14 373–386. 10.4103/1673-5374.245462 30539801 PMC6334604

[B67] Guerrero B. L. Sicotte N. L. (2020). Microglia in multiple sclerosis: friend or foe? *Front. Immunol.* 11:374. 10.3389/fimmu.2020.00374 32265902 PMC7098953

[B68] Hara T. Nakamura K. Matsui M. Yamamoto A. Nakahara Y. Suzuki-Migishima R. (2006). Suppression of basal autophagy in neural cells causes neurodegenerative disease in Mice. *Nature* 441 885–889. 10.1038/nature04724 16625204

[B69] Harris J. De Haro S. A. Master S. S. Keane J. Roberts E. A. Delgado M. (2007). T helper 2 cytokines inhibit autophagic control of intracellular mycobacterium tuberculosis. *Immunity* 27 505–517. 10.1016/j.immuni.2007.07.022 17892853

[B70] Harris J. Keane J. (2010). How tumour necrosis factor blockers interfere with tuberculosis immunity. *Clin. Exp. Immunol.* 161 1–9. 10.1111/j.1365-2249.2010.04146.x 20491796 PMC2940142

[B71] Hassanpour M. Hajihassani F. Hiradfar A. Aghamohammadzadeh N. Rahbarghazi R. Safaie N. (2020). Real-state of autophagy signaling pathway in neurodegenerative disease; focus on multiple sclerosis. *J. Inflamm.* 17 1–8. 10.1186/s12950-020-0237-8 32082082 PMC7014934

[B72] Hauser S. L. Waubant E. Arnold D. L. Vollmer T. Antel J. Fox R. J. (2008). B-cell depletion with rituximab in relapsing-remitting multiple sclerosis. *N. Engl. J. Med.* 358 676–688. 10.1056/NEJMoa0706383 18272891

[B73] He C. Klionsky D. J. (2009). Regulation mechanisms and signaling pathways of autophagy. *Ann. Rev. Genet.* 43 67–93. 10.1146/annurev-genet-102808-114910 19653858 PMC2831538

[B74] He Y. She H. Zhang T. Xu H. Cheng L. Yepes M. (2018). P38 MAPK inhibits autophagy and promotes microglial inflammatory responses by phosphorylating ULK1. *J. Cell Biol.* 217 315–328. 10.1083/jcb.201701049 29196462 PMC5748971

[B75] Heppner F. L. Greter M. Marino D. Falsig J. Raivich G. Hövelmeyer N. (2005). Experimental autoimmune encephalomyelitis repressed by microglial paralysis. *Nat. Med.* 11 146–152. 10.1038/nm1177 15665833

[B76] Hill R. A. Li A. M. Grutzendler J. (2018). Lifelong cortical myelin plasticity and age-related degeneration in the live mammalian brain. *Nat. Neurosci.* 21 683–695. 10.1038/s41593-018-0120-6 29556031 PMC5920745

[B77] Hirotani M. Niino M. Fukazawa T. Kikuchi S. Yabe I. Hamada S. (2010). Decreased IL-10 production mediated by toll-like receptor 9 in B cells in multiple sclerosis. *J. Neuroimmunol.* 221 95–100. 10.1016/j.jneuroim.2010.02.012 20227772

[B78] Hubbard V. M. Valdor R. Patel B. Singh R. Cuervo A. M. Macian F. (2010). Macroautophagy regulates energy metabolism during effector T cell activation. *J. Immunol.* 185 7349–7357. 10.4049/jimmunol.1000576 21059894 PMC3046774

[B79] Igci M. Baysan M. Yigiter R. Ulasli M. Geyik S. Bayraktar R. (2016). Gene expression profiles of autophagy-related genes in multiple sclerosis. *Gene* 588 38–46. 10.1016/j.gene.2016.04.042 27125224

[B80] Ireland J. M. Unanue E. R. (2011). Autophagy in antigen-presenting cells results in presentation of citrullinated peptides to CD4 T cells. *J. Exp. Med.* 208 2625–2632. 10.1084/jem.20110640 22162830 PMC3244027

[B81] Jagessar S. A. Holtman I. R. Hofman S. Morandi E. Heijmans N. Laman J. D. (2016). Lymphocryptovirus infection of nonhuman primate B cells converts destructive into productive processing of the pathogenic CD8 T cell epitope in myelin oligodendrocyte glycoprotein. *J. Immunol.* 197 1074–1088. 10.4049/jimmunol.1600124 27412414 PMC4974490

[B82] Jakimovski D. Weinstock-Guttman B. Ramanathan M. Kolb C. Hojnacki D. Minagar A. (2017). Ocrelizumab: a B-cell depleting therapy for multiple sclerosis. *Expert Opin. Biol. Ther.* 17 1163–1172. 10.1080/14712598.2017.1347632 28658986

[B83] Jang S. Y. Shin Y. K. Park S. Y. Park J. Y. Lee H. J. Yoo Y. H. (2016). Autophagic myelin destruction by schwann cells during wallerian degeneration and segmental demyelination. *Glia* 64 730–742. 10.1002/glia.22957 26712109

[B84] Jia W. He Y. W. (2011). Temporal regulation of intracellular organelle homeostasis in T lymphocytes by autophagy. *J. Immunol.* 186 5313–5322. 10.4049/jimmunol.1002404 21421856

[B85] Jin M. M. Wang F. Qi D. Liu W. W. Gu C. Mao C. J. (2018). A critical role of autophagy in regulating microglia polarization in neurodegeneration. *Front. Aging Neurosci.* 10:378. 10.3389/fnagi.2018.00378 30515090 PMC6256089

[B86] Joe E. H. Choi D. J. An J. Eun J. H. Jou I. Park S. (2018). Astrocytes, microglia, and Parkinson’s disease. *Exp. Neurobiol.* 27 77–87. 10.5607/en.2018.27.2.77 29731673 PMC5934545

[B87] Keller C. W. Lünemann J. D. (2017). Autophagy and autophagy-related proteins in CNS autoimmunity. *Front. Immunol.* 8:165. 10.3389/fimmu.2017.00165 28289410 PMC5326760

[B88] Keller C. W. Sina C. Kotur M. B. Ramelli G. Mundt S. Quast I. (2017). ATG-dependent phagocytosis in dendritic cells drives myelin-specific CD4+ T cell pathogenicity during CNS inflammation. *Proc. Natl. Acad. Sci. U.S.A.* 114 E11228–E11237. 10.1073/pnas.1713664114 29233943 PMC5748192

[B89] Kierdorf K. Erny D. Goldmann T. Sander V. Schulz C. Perdiguero E. G. (2013). Microglia emerge from erythromyeloid precursors via Pu.1-and Irf8-dependent pathways. *Nat. Neurosci.* 16 273–280. 10.1038/nn.3318 23334579

[B90] Kim H. J. Cho M. H. Shim W. H. Kim J. K. Jeon E. Y. Kim D. H. (2017). Deficient autophagy in microglia impairs synaptic pruning and causes social behavioral defects. *Mol. Psychiatry* 22 1576–1584. 10.1038/mp.2016.103 27400854 PMC5658669

[B91] Kirkin V. Rogov V. V. (2019). A diversity of selective autophagy receptors determines the specificity of the autophagy pathway. *Mol. Cell* 76 268–285. 10.1016/j.molcel.2019.09.005 31585693

[B92] Komatsu M. Waguri S. Chiba T. Murata S. Iwata J. Tanida I. (2006). Loss of autophagy in the central nervous system causes neurodegeneration in mice. *Nature* 441 880–884. 10.1038/nature04723 16625205

[B93] Komatsu M. Waguri S. Koike M. Sou Y. S. Ueno T. Hara T. (2007). Homeostatic levels of P62 control cytoplasmic inclusion body formation in autophagy-deficient mice. *Cell* 131 1149–1163. 10.1016/j.cell.2007.10.035 18083104

[B94] Kovacs J. R. Li C. Yang Q. Li G. Garcia I. G. Ju S. (2012). Autophagy promotes T-Cell survival through degradation of proteins of the cell death machinery. *Cell Death Differ.* 19 144–152. 10.1038/cdd.2011.78 21660048 PMC3252822

[B95] Lahiri V. Hawkins W. D. Klionsky D. J. (2019). Watch what you (self-) eat: autophagic mechanisms that modulate metabolism. *Cell Metab.* 29 803–826. 10.1016/j.cmet.2019.03.003 30943392 PMC6450419

[B96] Lai S. C. Devenish R. J. (2012). LC3-associated phagocytosis (LAP): connections with host autophagy. *Cells* 1 396–408. 10.3390/cells1030396 24710482 PMC3901117

[B97] Lampron A. Larochelle A. Laflamme N. Préfontaine P. Plante M. M. Sánchez M. G. (2015). Inefficient clearance of myelin debris by microglia impairs remyelinating processes. *J. Exp. Med.* 212 481–495. 10.1084/jem.20141656 25779633 PMC4387282

[B98] Lassmann H. Brück W. Lucchinetti C. F. (2007). The Immunopathology of Multiple Sclerosis: an Overview. *Brain Pathol.* 17 210–218. 10.1111/j.1750-3639.2007.00064.x 17388952 PMC8095582

[B99] Law A. H. Lee D. C. Yuen K. Y. Peiris M. Lau A. S. (2010). Cellular response to influenza virus infection: a potential role for autophagy in CXCL10 and interferon-alpha induction. *Cell. Mol. Immunol.* 7 263–270. 10.1038/cmi.2010.25 20473322 PMC4003230

[B100] Lee H. Y. Mattei L. M. Steinberg B. E. Alberts P. Lee Y. H. Chervonsky A. (2010). In vivo requirement for Atg5 in antigen presentation by dendritic cells. *Immunity* 32 227–239. 10.1016/j.immuni.2009.12.006 20171125 PMC2996467

[B101] Lee S. J. Cho Koh J. Y. (2009). Oxidative injury triggers autophagy in astrocytes: the role of endogenous zinc. *Glia* 57 1351–1361. 10.1002/glia.20854 19229997

[B102] Lehmann Horn K. Kronsbein H. C. Weber M. S. (2013). Targeting B cells in the treatment of multiple sclerosis: recent advances and remaining challenges. *Therapeut. Adv. Neurol. Disord.* 6 161–173. 10.1177/1756285612474333 23634189 PMC3625013

[B103] Levine B. Kroemer G. (2019). Biological functions of autophagy genes: a disease perspective. *Cell* 176 11–42. 10.1016/j.cell.2018.09.048 30633901 PMC6347410

[B104] Levine B. Mizushima N. Virgin H. W. (2011). Autophagy in immunity and inflammation. *Nature* 469 323–335. 10.1038/nature09782 21248839 PMC3131688

[B105] Li C. Capan E. Zhao Y. Zhao J. Stolz D. Watkins S. C. (2006). Autophagy is induced in CD4 + T Cells and important for the growth factor-withdrawal cell death. *J. Immunol.* 177 5163–5168. 10.4049/jimmunol.177.8.5163 17015701

[B106] Li R. Patterson K. R. Bar-Or A. (2018). Reassessing B cell contributions in multiple sclerosis. *Nat. Immunol.* 19 696–707. 10.1038/s41590-018-0135-x 29925992

[B107] Li W. W. Li J. Bao J. K. (2012). Microautophagy: lesser-known self-eating. *Cell. Mol. Life Sci.* 69 1125–1136. 10.1007/s00018-011-0865-5 22080117 PMC11114512

[B108] Li W. W. Setzu A. Zhao C. Franklin R. J. (2005). Minocycline-mediated inhibition of microglia activation impairs oligodendrocyte progenitor cell responses and remyelination in a non-immune model of demyelination. *J. Neuroimmunol.* 158 58–66. 10.1016/j.jneuroim.2004.08.011 15589038

[B109] Liang P. Le W. (2015). Role of autophagy in the pathogenesis of multiple sclerosis. *Neurosci. Bull.* 31 435–444. 10.1007/s12264-015-1545-5 26254059 PMC5563716

[B110] Lieberman O. J. McGuirt A. F. Tang G. Sulzer D. (2019). Roles for neuronal and glial autophagy in synaptic pruning during development. *Neurobiol. Dis.* 122 49–63. 10.1016/j.nbd.2018.04.017 29709573 PMC6204314

[B111] Lin N. Y. Beyer C. Gießl A. Kireva T. Scholtysek C. Uderhardt S. (2013). Autophagy regulates TNFα-mediated joint destruction in experimental arthritis. *Ann. Rheum. Dis.* 72 761–768. 10.1136/annrheumdis-2012-201671 22975756

[B112] Liu X. Tian F. Wang S. Wang F. Xiong L. (2018). Astrocyte autophagy flux protects neurons against oxygen-glucose deprivation and ischemic/reperfusion injury. *Rejuvenat. Res.* 21 405–415. 10.1089/rej.2017.1999 29125039

[B113] Livingston P. G. Kurane I. Ennis F. A. (1997). Use of epstein-barr virus-transformed, autologous B-Lymphoblastoid cells as antigen-presenting cells for establishment and maintenance of dengue virus-specific, human cytotoxic T lymphocyte clones. *J. Virol. Methods* 67 77–84. 10.1016/S0166-0934(97)00082-7 9274820

[B114] Lloyd A. F. Davies C. L. Miron V. E. (2017). Microglia: origins, homeostasis, and roles in myelin repair. *Curr. Opin. Neurobiol.* 47 113–120. 10.1016/j.conb.2017.10.001 29073528

[B115] Lovas G. Szilágyi N. Majtényi K. Palkovits M. Komoly S. (2000). Axonal changes in chronic demyelinated cervical spinal cord plaques. *Brain* 123 308–317. 10.1093/brain/123.2.308 10648438

[B116] Luo C. Jian C. Liao Y. Huang Q. Wu Y. Liu X. (2017). The role of microglia in multiple sclerosis. *Neuropsych. Dis. Treat.* 13 1661–1667. 10.2147/NDT.S140634 28721047 PMC5499932

[B117] Macian F. (2019). Autophagy in T cell function and aging. *Front. Cell Dev. Biol.* 7:2013. 10.3389/fcell.2019.00213 31632966 PMC6783498

[B118] Malta C. D. Fryer J. D. Settembre C. Ballabio A. (2012). Astrocyte dysfunction triggers neurodegeneration in a lysosomal storage disorder. *Proc. Natl. Acad. Sci. U.S.A.* 109 E2334–E2342. 10.1073/pnas.1209577109 22826245 PMC3435187

[B119] Martinez-Vicente M. Cuervo A. M. (2007). Autophagy and neurodegeneration: when the cleaning crew goes on strike. *Lancet Neurol.* 6 352–361. 10.1016/S1474-4422(07)70076-5 17362839

[B120] Meikle L. Pollizzi K. Egnor A. Kramvis I. Lane H. Sahin M. (2008). Response of a neuronal model of tuberous sclerosis to mammalian target of rapamycin (MTOR) inhibitors: effects on MTORC1 and Akt signaling lead to improved survival and function. *J. Neurosci.* 28 5422–5432. 10.1523/JNEUROSCI.0955-08.2008 18495876 PMC2633923

[B121] Melief J. Koning N. Schuurman K. G. Van De Garde M. D. Smolders J. Hoek R. M. (2012). Phenotyping Primary human microglia: tight regulation of LPS responsiveness. *Glia* 60 1506–1517. 10.1002/glia.22370 22740309

[B122] Melief J. Schuurman K. G. Van de Garde M. D. Smolders J. Van Eijk M. Hamann J. (2013). Microglia in normal appearing white matter of multiple sclerosis are alerted but immunosuppressed. *Glia* 61 1848–1861. 10.1002/glia.22562 24014207

[B123] Menzies F. M. Fleming A. Caricasole A. Bento C. F. Andrews S. P. Ashkenazi A. (2017). Autophagy and neurodegeneration: pathogenic mechanisms and therapeutic opportunities. *Neuron* 93 1015–1034. 10.1016/j.neuron.2017.01.022 28279350

[B124] Miller B. C. Zhao Z. Stephenson L. M. Cadwell K. Pua H. H. Lee H. K. (2008). The autophagy gene ATG5 plays an essential role in B lymphocyte development. *Autophagy* 4 309–314. 10.4161/auto.5474 18188005

[B125] Miron V. E. Boyd A. Zhao J. W. Yuen T. J. Ruckh J. M. Shadrach J. L. (2013). M2 microglia and macrophages drive oligodendrocyte differentiation during CNS remyelination. *Nat. Neurosci.* 16 1211–1218. 10.1038/nn.3469 23872599 PMC3977045

[B126] Mizushima N. Levine B. Cuervo A. M. Klionsky D. J. (2008). Autophagy fights disease through cellular self-digestion. *Nature* 451 1069–1075. 10.1038/nature06639 18305538 PMC2670399

[B127] Mohammad M. G. Hassanpour M. Tsai V. W. Li H. Ruitenberg M. J. Booth D. W. (2013). Dendritic Cells and multiple sclerosis: disease, tolerance and therapy. *Int. J. Mol. Sci.* 14 547–562. 10.3390/ijms14010547 23271370 PMC3565281

[B128] Morandi E. Jagessar S. A. t Hart B. A. Bruno B. (2017). EBV infection empowers human B cells for autoimmunity: role of autophagy and relevance to multiple sclerosis. *J. Immunol.* 199 435–448. 10.4049/jimmunol.1700178 28592428

[B129] Morselli E. Mariño G. Bennetzen M. V. Eisenberg T. Megalou E. Schroeder S. (2011). Spermidine and resveratrol induce autophagy by distinct pathways converging on the acetylproteome. *J. Cell Biol.* 192 615–629. 10.1083/jcb.201008167 21339330 PMC3044119

[B130] Motori E. Puyal J. Toni N. Ghanem A. Angeloni C. Malaguti M. (2013). Inflammation-induced alteration of astrocyte mitochondrial dynamics requires autophagy for mitochondrial network maintenance. *Cell Metab.* 18 844–859. 10.1016/j.cmet.2013.11.005 24315370

[B131] Mulero P. Midaglia L. Montalban X. (2018). Ocrelizumab: a new milestone in multiple sclerosis therapy. *Therap. Adv. Neurol. Disord.* 11 1–6. 10.1177/1756286418773025 29774057 PMC5952271

[B132] Muller S. Brun S. René F. de Sèze J. Loeffler J. P. Jeltsch-David H. (2017). Autophagy in neuroinflammatory diseases. *Autoimmun. Rev.* 16 856–874. 10.1016/j.autrev.2017.05.015 28572049

[B133] Münz C. (2016). Autophagy proteins in antigen processing for presentation on MHC molecules. *Immunol. Rev.* 272 17–27. 10.1111/imr.12422 27319339

[B134] Nakatogawa H. (2020). Mechanisms governing autophagosome biogenesis. *Nat. Rev. Mol. Cell Biol.* 21 439–458. 10.1038/s41580-020-0241-0 32372019

[B135] Nave K. A. Werner H. B. (2014). Myelination of the Nervous system: mechanisms and functions. *Annu. Rev. Cell Dev. Biol.* 30 503–533. 10.1146/annurev-cellbio-100913-013101 25288117

[B136] Nissen J. C. Thompson K. K. West B. L. Tsirka S. E. (2018). Csf1R inhibition attenuates experimental autoimmune encephalomyelitis and promotes recovery. *Exp. Neurol.* 307 24–36. 10.1016/j.expneurol.2018.05.021 29803827 PMC6380683

[B137] Nixon R. A. (2013). The role of autophagy in neurodegenerative disease. *Nat. Med.* 19 983–997. 10.1038/nm.3232 23921753

[B138] Nixon R. A. Yang D. S. (2011). Autophagy failure in Alzheimer’s disease-locating the primary defect. *Neurobiol. Dis.* 43 38–45. 10.1016/j.nbd.2011.01.021 21296668 PMC3096679

[B139] Nuyts A. H. Lee W. P. Bashir-Dar R. Berneman Z. N. Cools N. (2013). Dendritic cells in multiple sclerosis: key players in the Immunopathogenesis, key players for new cellular immunotherapies? *Mult. Scler. J.* 19 995–1002. 10.1177/1352458512473189 23369893

[B140] Orihuela R. McPherson C. A. Harry G. J. (2016). Microglial M1/M2 polarization and metabolic states. *Br. J. Pharmacol.* 173 649–665. 10.1111/bph.13139 25800044 PMC4742299

[B141] Paludan C. Schmid D. Landthaler M. Vockerodt M. Kube D. Tuschl T. (2005). Endogenous MHC class II processing of a viral nuclear antigen after autophagy. *Science* 307 593–596. 10.1126/science.1104904 15591165

[B142] Parekh V. V. Wu L. Kelli L. Janice A. B. Jennifer A. W. Olivares-Villagómez G. D. (2013). Impaired autophagy, defective T cell homeostasis, and a wasting syndrome in mice with a T cell–specific deletion of Vps34. *J. Immunol.* 190 5086–5101. 10.4049/jimmunol.1202071 23596309 PMC3646937

[B143] Park H. J. Lee S. J. Kim S. H. Han J. Bae J. Kim S. J. (2011). IL-10 Inhibits the starvation induced autophagy in macrophages via class i phosphatidylinositol 3-kinase (PI3K) pathway. *Mol. Immunol.* 48 720–727. 10.1016/j.molimm.2010.10.020 21095008

[B144] Patsopoulos N. A. Baranzini S. E. Santaniello A. Shoostari P. Cotsapas C. Wong G. (2019). Multiple sclerosis genomic map implicates peripheral immune cells and microglia in susceptibility. *Science* 365:6460. 10.1126/science.aav7188 31604244 PMC7241648

[B145] Paunovic V. Petrovic I. V. Milenkovic M. Janjetovic K. Pravica V. Dujmovic I. (2018). Autophagy-independent increase of ATG5 expression in T cells of multiple sclerosis patients. *J. Neuroimmunol.* 319 100–105. 10.1016/j.jneuroim.2018.03.001 29548704

[B146] Pengo N. Scolari M. Oliva L. Milan E. Mainoldi F. Raimondi A. (2013). Plasma cells require autophagy for sustainable immunoglobulin production. *Nat. Immunol.* 14 298–305. 10.1038/ni.2524 23354484

[B147] Plaza-Zabala A. Sierra-Torre V. Sierra A. (2017). Autophagy and microglia: novel partners in neurodegeneration and aging. *Int. J. Mol. Sci.* 18:598. 10.3390/ijms18030598 28282924 PMC5372614

[B148] Ponath G. Park C. Pitt D. (2018). The Role of astrocytes in multiple sclerosis. *Front. Immunol.* 9:217. 10.3389/fimmu.2018.00217 29515568 PMC5826071

[B149] Ponomarev E. D. Leah P. S. Maresz K. Bonnie N. D. (2005). Microglial cell activation and proliferation precedes the onset of CNS autoimmunity. *J. Neurosci. Res.* 81 374–389. 10.1002/jnr.20488 15959904

[B150] Prineas J. W. Kwon E. E. Sook E. Cho L. R. Sharer M. H. Barnett E. L. (2001). Immunopathology of secondary-progressive multiple sclerosis. *Ann. Neurol.* 50 646–657. 10.1002/ana.1255 11706971

[B151] Pua H. H. Dzhagalov I. Chuck M. Mizushima N. He Y. W. (2007). A critical role for the autophagy gene Atg5 in T cell survival and proliferation. *J. Exp. Med.* 204 25–31. 10.1084/jem.20061303 17190837 PMC2118420

[B152] Pua H. H. Guo J. Komatsu M. He Y.-W. (2009). Autophagy Is essential for mitochondrial clearance in mature T lymphocytes. *J. Immunol.* 182 4046–4055. 10.4049/jimmunol.0801143 19299702

[B153] Pua H. H. He Y. W. (2007). Maintaining T lymphocyte homeostasis: another duty of autophagy. *Autophagy* 3 266–267. 10.4161/auto.3908 17329964

[B154] Puente C. Hendrickson R. C. Jiang X. (2016). Nutrient-regulated phosphorylation of ATG13 inhibits starvation-induced autophagy. *J. Biol. Chem.* 291 6026–6035. 10.1074/jbc.M115.689646 26801615 PMC4786734

[B155] Puleston D. J. Simon A. K. (2014). Autophagy in the immune system. *Immunology* 141 1–8. 10.1111/imm.12165 23991647 PMC3893844

[B156] Qian M. Fang X. Wang X. (2017). Autophagy and inflammation. *Clin. Trans. Med.* 6:24. 10.1186/s40169-017-0154-5 28748360 PMC5529308

[B157] Ramaglia V. Hughes T. R. Donev R. M. Ruseva M. M. Wu X. Huitinga I. (2012). C3-dependent mechanism of microglial priming relevant to multiple sclerosis. *Proc. Natl. Acad. Sci. U.S.A.* 109 965–970. 10.1073/pnas.1111924109 22219359 PMC3271873

[B158] Rangaraju S. Verrier J. D. Madorsky I. Nicks J. Dunn W. A. Notterpek L. (2010). Rapamycin activates autophagy and improves myelination in explant cultures from neuropathic mice. *J. Neurosci.* 30 11388–11397. 10.1523/JNEUROSCI.1356-10.2010 20739560 PMC3478092

[B159] Rathmell J. C. (2012). Metabolism and autophagy in the immune system: immunometabolism comes of age. *Immunol. Rev.* 249 5–13. 10.1111/j.1600-065X.2012.01158.x 22889211 PMC3576876

[B160] Ravikumar B. Moreau K. Jahreiss L. Puri C. Rubinsztein D. C. (2010). Plasma membrane contributes to the formation of pre-autophagosomal structures. *Nat. Cell Biol.* 12 747–757. 10.1038/ncb2078 20639872 PMC2923063

[B161] Ravikumar B. Vacher C. Berger Z. Davies J. E. Luo S. Oroz L. G. (2004). Inhibition of MTOR induces autophagy and reduces toxicity of polyglutamine expansions in fly and mouse models of huntington disease. *Nat. Genet.* 36 585–595. 10.1038/ng1362 15146184

[B162] Rezai-Zadeh K. Gate D. Town T. (2009). CNS infiltration of peripheral immune cells: D-day for neurodegenerative disease? *J. Neuroim. Pharmacol.* 4 462–475. 10.1007/s11481-009-9166-2 19669892 PMC2773117

[B163] Rubinsztein D. C. Bento C. F. Deretic V. (2015). Therapeutic targeting of autophagy in neurodegenerative and infectious diseases. *J. Exp. Med.* 212 979–990. 10.1084/jem.20150956 26101267 PMC4493419

[B164] Saitoh T. Fujita N. Jang M. H. Uematsu S. Yang B. G. Satoh T. (2008). Loss of the autophagy protein Atg16L1 enhances endotoxin-induced IL-1β production. *Nature* 456 264–268. 10.1038/nature07383 18849965

[B165] Salminen A. Kaarniranta K. Kauppinen A. (2013). Beclin 1 interactome controls the crosstalk between apoptosis, autophagy and inflammasome activation: impact on the aging process. *Ageing Res. Rev.* 12 520–534. 10.1016/j.arr.2012.11.004 23220384

[B166] Salou M. Nicol B. Garcia A. Laplaud D. A. (2015). Involvement of CD8+ T cells in multiple sclerosis. *Front. Immunol.* 6:604. 10.3389/fimmu.2015.00604 26635816 PMC4659893

[B167] Sanjuan M. A. Dillon C. P. Stephen W. G. Moshiach T. S. Dorsey F. Connell S. (2007). Toll-like receptor signalling in macrophages links the autophagy pathway to phagocytosis. *Nature* 450 1253–1257. 10.1038/nature06421 18097414

[B168] Sawcer S. Hellenthal G. Pirinen M. Spencer C. C. A. Patsopoulos N. A. Moutsianas L. (2011). Genetic risk and a primary role for cell-mediated immune mechanisms in multiple sclerosis. *Nature* 476 214–219. 10.1038/nature10251 21833088 PMC3182531

[B169] Schmid D. Park C. G. Hartl C. A. Subedi N. Cartwright A. N. Puerto R. B. (2017). T cell-targeting nanoparticles focus delivery of immunotherapy to improve antitumor immunity. *Nat. Commun.* 8 1–11. 10.1038/s41467-017-01830-8 29170511 PMC5700944

[B170] Schmid D. Pypaert M. Münz C. (2007). Antigen-loading compartments for major histocompatibility complex class II Molecules continuously receive input from autophagosomes. *Immunity* 26 79–92. 10.1016/j.immuni.2006.10.018 17182262 PMC1805710

[B171] Schulz C. Perdiguero E. G. Chorro L. Szabo-Rogers H. Cagnard N. Kierdorf K. (2012). A lineage of myeloid cells independent of Myb and hematopoietic stem cells. *Science* 335 86–90. 10.1126/science.1219179 22442384

[B172] Shao B. Z. Wei W. Ke P. Xu Z. Q. Zhou J. X. Liu C. (2014). Activating cannabinoid receptor 2 alleviates pathogenesis of experimental autoimmune encephalomyelitis via activation of autophagy and inhibiting NLRP3 inflammasome. *CNS Neurosci. Therapeut.* 20 1021–1028. 10.1111/cns.12349 25417929 PMC6492996

[B173] Shi C. S. Shenderov K. Huang N. N. Kabat J. Abu-Asab M. Fitzgerald K. A. (2012). Activation of autophagy by inflammatory signals limits IL-1β production by targeting ubiquitinated inflammasomes for destruction. *Nat. Immunol.* 13 255–263. 10.1038/ni.2215 22286270 PMC4116819

[B174] Shoji-Kawata S. Sumpter R. Leveno M. Campbell G. R. Zou Z. Kinch L. (2013). Identification of a candidate therapeutic autophagy-inducing peptide. *Nature* 494 201–206. 10.1038/nature11866 23364696 PMC3788641

[B175] Skripuletz T. Hackstette D. Bauer K. Gudi V. Pul R. Voss E. (2013). Astrocytes regulate myelin clearance through recruitment of microglia during cuprizone-induced demyelination. *Brain* 136 147–167. 10.1093/brain/aws262 23266461

[B176] Smith C. M. Mayer J. A. Duncan I. D. (2013). Autophagy promotes oligodendrocyte survival and function following dysmyelination in a long-lived myelin mutant. *J. Neurosci.* 33 8088–8100. 10.1523/JNEUROSCI.0233-13.2013 23637198 PMC4128639

[B177] Sofroniew M. V. Vinters H. V. (2010). Astrocytes: biology and pathology. *Acta Neuropathol.* 119 7–35. 10.1007/s00401-009-0619-8 20012068 PMC2799634

[B178] Sospedra M. (2018). B cells in multiple sclerosis. *Curr. Opin. Neurol.* 31 256–262. 10.1097/WCO.000000000000563 29629941

[B179] Stamatakou E. Wróbel L. Hill S. M. Puri C. Son S. M. Fujimaki M. (2020). Mendelian neurodegenerative disease genes involved in autophagy. *Cell Discov.* 6:158. 10.1038/s41421-020-0158-y 32377374 PMC7198619

[B180] Stavoe A. K. H. Holzbaur E. L. F. (2019). Autophagy in neurons. *Ann. Rev. Cell Dev. Biol.* 35 477–500. 10.1146/annurev-cellbio-100818-125242 31340124 PMC6996145

[B181] Stoffels J. M. J. De Jonge J. C. Stancic M. Nomden A. Van Strien M. E. Šišková D. M. Z. (2013). Fibronectin aggregation in multiple sclerosis lesions impairs remyelination. *Brain* 136 116–131. 10.1093/brain/aws313 23365094

[B182] Su P. Zhang J. Wang D. Zhao F. Cao Z. Aschner M. (2016). The role of autophagy in modulation of neuroinflammation in microglia. *Neuroscience* 319 155–167. 10.1016/j.neuroscience.2016.01.035 26827945

[B183] ’t Hart B. A. Kap Y. S. Morandi E. Laman J. D. Gran B. (2016). EBV infection and multiple sclerosis: lessons from a marmoset model. *Trends Mol. Med.* 22 1012–1024. 10.1016/j.molmed.2016.10.007 27836419

[B184] Thorley-Lawson D. A. Mann K. P. (1985). Early events in epstein-barr virus infection provide a model for b cell activation. *J. Exp. Med.* 162 45–59. 10.1084/jem.162.1.45 2989413 PMC2187697

[B185] Trapp B. D. Nave K. A. (2008). Multiple sclerosis: an immune or neurodegenerative disorder? *Ann. Rev. Neurosci.* 31 247–269. 10.1146/annurev.neuro.30.051606.094313 18558855

[B186] Trapp B. D. Peterson J. Ransohoff R. M. Rudick R. Mörk S. Bö L. (1998). Axonal transection in the lesions of multiple sclerosis. *New Engl. J. Med.* 338 278–285. 10.1056/NEJM199801293380502 9445407

[B187] Tsunoda I. Fujinami R. S. (2002). Inside-out versus outside-in models for virus induced demyelination: axonal damage triggering demyelination. *Springer Semin. Immunopathol.* 24 105–125. 10.1007/s00281-002-0105-z 12503060 PMC7079941

[B188] Tsunoda I. Kuang L. Q. Libbey J. E. Fujinami R. S. (2003). Axonal injury heralds virus-induced demyelination. *Am. J. Pathol.* 162 1259–1269. 10.1016/S0002-9440(10)63922-3 12651618 PMC1851221

[B189] van Beek N. Klionsky D. Reggiori F. (2018). Genetic aberrations in macroautophagy genes leading to diseases. *Biochim. Biophys. Acta Mol. Cell Res.* 1865 803–816. 10.1016/j.bbamcr.2018.03.002 29524522

[B190] Varshney P. Saini N. (2018). PI3K/AKT/MTOR activation and autophagy inhibition plays a key role in increased cholesterol during IL-17A mediated inflammatory response in psoriasis. *Biochim. Biophys. Acta Mol. Basis Dis.* 1864 1795–1803. 10.1016/j.bbadis.2018.02.003 29432814

[B191] von Büdingen H. C. Palanichamy A. Lehmann-Horn K. Michel B. A. Zamvil S. S. (2015). Update on the autoimmune pathology of multiple sclerosis: B-cells as disease-drivers and therapeutic targets. *Eur. Neurol.* 73 238–246. 10.1159/000377675 25824054 PMC4401636

[B192] Voß E. V. Škuljec J. Gudi V. Skripuletz T. Pul R. Trebst C. (2012). Characterisation of microglia during De- and remyelination: can they create a repair promoting environment? *Neurobiol. Dis.* 45 519–528. 10.1016/j.nbd.2011.09.008 21971527

[B193] Wang J. Wang J. Wang J. Yang B. Weng Q. He Q. (2019). Targeting microglia and macrophages: a potential treatment strategy for multiple sclerosis. *Front. Pharmacol.* 10:286. 10.3389/fphar.2019.00286 30967783 PMC6438858

[B194] Wang J. L. Xu C. J. (2020). Astrocytes autophagy in aging and neurodegenerative disorders. *Biomed. Pharmacother.* 122:109691. 10.1016/j.biopha.2019.109691 31786465

[B195] Wang S. Li B. Qiao H. Lv X. Liang Q. Shi Z. (2014). Autophagy−related Gene Atg5 is essential for astrocyte differentiation in the developing mouse cortex. *EMBO Rep.* 15 1053–1061. 10.15252/embr.201338343 25227738 PMC4253845

[B196] Werneburg S. Jung J. Kunjamma R. B. Ha S. K. Luciano N. J. Willis C. M. (2020). Targeted complement inhibition at synapses prevents microglial synaptic engulfment and synapse loss in demyelinating disease. *Immunity* 52 167–182.e7. 10.1016/j.immuni.2019.12.004 31883839 PMC6996144

[B197] Wolswijk G. (2000). Oligodendrocyte Survival, loss and birth in lesions of chronic-stage multiple sclerosis. *Brain* 123 105–115. 10.1093/brain/123.1.105 10611125

[B198] Wolswijk G. (2002). Oligodendrocyte precursor cells in the demyelinated multiple sclerosis spinal cord. *Brain* 125 338–349. 10.1093/brain/awf031 11844734

[B199] Wong E. Cuervo A. M. (2010). Autophagy gone awry in neurodegenerative diseases. *Nat. Neurosci.* 13 805–811. 10.1038/nn.2575 20581817 PMC4038747

[B200] Wong Y. C. Holzbaur E. L. F. (2014). Optineurin Is an autophagy receptor for damaged mitochondria in parkin-mediated mitophagy that is disrupted by an ALS-linked mutation. *Proc. Natl. Acad. Sci. U.S.A.* 111 E4439–E4448. 10.1073/pnas.1405752111 25294927 PMC4210283

[B201] Yang Z. Goronzy J. J. Weyand C. M. (2015). Autophagy in autoimmune disease. *J. Mol. Med.* 93 707–717. 10.1007/s00109-015-1297-8 26054920 PMC4486076

[B202] Yin H. Wu H. Chen Y. Zhang J. Zheng M. Chen G. (2018). The therapeutic and pathogenic role of autophagy in autoimmune diseases. *Front. Immunol.* 9:1512. 10.3389/fimmu.2018.01512 30108582 PMC6080611

[B203] Yogev N. Frommer F. Lukas D. Kautz-Neu K. Karram K. Ielo D. (2012). Dendritic Cells ameliorate autoimmunity in the cns by controlling the homeostasis of PD-1 receptor+ regulatory T cells. *Immunity* 37 264–275. 10.1016/j.immuni.2012.05.025 22902234

[B204] Zachari M. Ganley I. G. (2017). The mammalian ULK1 complex and autophagy initiation. *Essays Biochem.* 61 585–596. 10.1042/EBC20170021 29233870 PMC5869855

[B205] Zeis T. Probst A. Steck A. J. Stadelmann C. Brück W. Schaeren-Wiemers N. (2009). Molecular changes in white matter adjacent to an active demyelinating lesion in early multiple sclerosis: molecular changes in MS periplaque white matter. *Brain Pathol.* 19 459–466. 10.1111/j.1750-3639.2008.00231.x 19016740 PMC8094783

[B206] Zhang F. Lin Y. A. Kannan S. Kannan R. M. (2016). Targeting specific cells in the brain with nanomedicines for CNS therapies. *J. Control. Release* 240 212–226. 10.1016/j.jconrel.2015.12.013 26686078 PMC4903089

[B207] Zhang H. Puleston D. J. Simon A. K. (2016). Autophagy and immune senescence. *Trends Mol. Med.* 22 671–686. 10.1016/j.molmed.2016.06.001 27395769

